# Efficacy and safety of thymosin combined with anticancer therapy for esophageal cancer: a systematic review and meta-analysis of randomized controlled trials

**DOI:** 10.3389/fimmu.2026.1812375

**Published:** 2026-05-29

**Authors:** Tao Sun, Ruping Zhao, Chunhui Zhang, Xishen Shan, Yaling Zhang, Yuling Zheng

**Affiliations:** 1 Department of Oncology, The First Affiliated Hospital of Henan University of Chinese Medicine, Zhengzhou, China; 2 Henan University of Chinese Medicine, Zhengzhou, China; 3 Paediatric Hospital, The First Affiliated Hospital of Henan University of Chinese Medicine, Zhengzhou, China; 4 The First Affiliated Hospital of Henan University of Chinese Medicine, Zhengzhou, China

**Keywords:** chemotherapy, esophageal cancer (EC), GRADE, radiotherapy, thymosin, TSA

## Abstract

**Objective:**

This study aims to comprehensively evaluate the clinical efficacy, impact on immune function, and safety of thymosin combined with anticancer therapy for esophageal cancer (EC) patients.

**Methods:**

We systematically searched Chinese and English databases to identify randomized controlled trials (RCTs) evaluating thymosin combined with standard anticancer therapy for EC. Meta-analysis was performed using Review Manager 5.4. Sensitivity analysis and publication bias assessment were conducted using Stata 18.0. TSA was further employed to validate the reliability of primary outcomes. Evidence quality was assessed using GRADE system.

**Results:**

A total of 18 RCTs (1,272 patients) were included. Meta-analysis demonstrated that compared with standard anticancer therapy alone, combined thymosin therapy significantly improved Objective Response Rate (ORR) (RR = 1.27, 95% CI: 1.17–1.39) and Disease Control Rate (DCR) (RR = 1.13, 95% CI: 1.07–1.19), and improved 1-year survival rate (RR = 1.36, 95% CI: 1.19–1.56), 2-year survival rate (RR = 1.47, 95% CI: 1.12–1.92), and 3-year survival rate (RR = 1.42, 95% CI: 1.07–1.90). Regarding immune function, the treatment group exhibited a higher CD3^+^ T lymphocyte ratio (CD3^+^%) (MD = 17.56, 95% CI: 13.63–21.50), CD4^+^ T lymphocyte ratio (CD4^+^%) (MD = 12.81, 95% CI: 10.76–14.87), CD4^+^/CD8^+^ ratio (MD = 0.71, 95% CI: 0.61 - 0.81), and natural killer (NK) cell level (MD = 4.02, 95% CI: 3.06–4.97) were significantly elevated. Regarding safety, combination therapy significantly reduced incidence of treatment-related adverse effects, including gastrointestinal reactions (nausea and vomiting) (RR = 0.69, 95% CI: 0.60–0.79), leukopenia (RR = 0.52, 95% CI: 0.43–0.63), radiation esophagitis (RR = 0.63, 95% CI: 0.44–0.90), and radiation pneumonitis (RR = 0.37, 95% CI: 0.22–0.62). However, methodological quality of included RCTs was limited, and a comprehensive safety assessment is limited by lack of data on thymosin-specific side effects. GRADE assessment indicates that the quality of evidence for most outcomes ranges from “very low” to “low.”

**Conclusion:**

Current evidence suggests that combination of thymosin with anticancer therapy for EC may enhance antitumor efficacy, improve patients’ immune function, and reduce toxicity associated with conventional treatments. However, given limited quality of RCTs and presence of publication bias, these conclusions require validation through additional large-scale, high-quality prospective research.

**Systematic review registration:**

https://www.crd.york.ac.uk/prospero/, identifier PROSPERO CRD420261282381.

## 1 Introduction

Esophageal cancer (EC) ranks among the most prevalent gastrointestinal malignancies globally, characterized by insidious onset, rapid progression, and poor prognosis (1, 2). Currently, for resectable EC, the primary treatment approach is a comprehensive regimen centered on surgical resection, supplemented by radiotherapy, chemotherapy, and immune checkpoint inhibitors (ICIs); whereas for advanced, recurrent, metastatic, or unresectable EC, the standard strategy involves a combination of radiotherapy, chemotherapy, targeted therapy, immunotherapy, and palliative care (3). Although these treatments have improved patient survival outcomes to some extent, their efficacy is often limited by treatment-related bone marrow suppression, lymphocyte depletion, and immune dysfunction (4–6). This treatment-induced immunosuppression not only increases infection risk but may also weaken the body’s own anti-tumor immune surveillance, potentially affecting long-term survival (7, 8). Therefore, alongside conventional antitumor therapies, exploring adjunctive strategies that protect and enhance patient immune function has become a critical research direction for improving EC treatment outcomes.

Immunomodulators, as therapeutic agents designed to restore or enhance the body’s immune response, show great promise in the field of comprehensive cancer treatment (9, 10). Among these, the thymosin family, particularly thymosin α1 (Thymalfasin), has garnered significant attention due to its unique immunomodulatory functions (11, 12). Thymosin, primarily secreted by thymic epithelial cells, promote T lymphocyte maturation, differentiation, and proliferation, enhance Th1-type cellular immune responses, and regulate cytokine balance (13). Preclinical and clinical studies indicate that thymosin can mitigate radiation and chemotherapy-induced immune damage, improve the quantity and function of immune cells (such as CD3^+^, CD4^+^ T cells, and NK cells), and potentially enhance antitumor effects by reshaping the tumor microenvironment (14–17). In clinical practice for various solid tumors including hepatocellular carcinoma and lung cancer, combination therapy with thymosin has demonstrated potential benefits in reducing infection rates, improving quality of life, and even prolonging survival (18–21).

In recent years, several randomized controlled trials (RCTs) have begun to evaluate the efficacy of thymosin α1 in combination with anticancer therapy for EC patients. Reported outcomes encompass multiple aspects including immune parameters, treatment toxicity, quality of life, and survival rates (22–24). However, these studies typically have small sample sizes, inconclusive findings, and lack systematic integration. While previous reviews have explored thymosin’s general role in cancer treatment (18), there remains a gap in systematic evaluations and meta-analyses specifically targeting EC—a highly prevalent malignancy with poor prognosis—to provide high-level evidence on the efficacy and safety of thymosin combined with anticancer therapy. Therefore, this study aims to conduct a systematic evaluation and meta-analysis of existing RCTs to comprehensively assess the efficacy and safety of thymosin combined with anticancer therapy for EC patients. This effort seeks to provide evidence-based medical guidance for clinical practice and clarify future research directions.

## 2 Methods

### 2.1 Study registration

This study was conducted in strict accordance with the PRISMA 2020 statement (25) (see **Supplementary Table 1**). To ensure transparency throughout the research process, protocol for this systematic review was registered with PROSPERO, with registration number CRD420261282381.

### 2.2 Search strategy

2 authors (T.S. and R.Z.) independently searched 8 electronic databases, covering the time period from inception to January 13, 2026, with no language restrictions. English databases included PubMed, Embase, Cochrane Library, and Web of Science; Chinese databases included China National Knowledge Infrastructure (CNKI), Wanfang Data, VIP Database, and SinoMed. The search strategy combined MeSH with free-text terms, using keywords such as “thymosin,” “thymalfasin,” “esophageal cancer,” and “esophageal malignant tumor.”

Additionally, we manually searched U.S. Clinical Trials Registry (https://clinicaltrials.gov/), China Clinical Trials Registry (https://www.chictr.org.cn/), and references of included studies and relevant review articles to minimize omissions. Detailed search strategies and results for each database are provided in **Supplementary Material S2**.

### 2.3 Eligibility criteria

The following criteria were established based on the PICOS principles:

#### 2.3.1 Inclusion criteria

1. P: Patients with pathologically confirmed EC, regardless of stage, treatment modality (surgery, chemoradiotherapy, etc.), or geographic location, were included.
2. I/C: Control group patients receive standard EC treatment regimens recommended by guidelines (including surgery, chemotherapy, radiotherapy, or immunotherapy) (26, 27); The experimental group received any form of thymosin preparation (e.g., thymosin α-1, thymopentin, thymosin injection) in combination with conventional anticancer therapy. Administration route, dosage, and treatment duration were unrestricted.
3. O: Primary outcomes: Objective Response Rate (ORR) and Disease Control Rate (DCR). ORR and DCR were assessed according to the Response Evaluation Criteria in Solid Tumors (RECIST) (28).

Secondary outcomes: 1) 1-year, 2-year, and 3-year survival rates. 2) Immunological parameters: including CD3^+^ T lymphocyte ratio (CD3^+^%), CD4^+^ T lymphocyte ratio (CD4^+^%), CD8^+^ T lymphocyte ratio (CD8^+^%), CD4^+^/CD8^+^ ratio, and natural killer (NK) cell level. 3) Improvement rate in Karnofsky Performance Status (KPS) score. 4) Incidence of treatment-related adverse effects: including gastrointestinal reactions (nausea and vomiting), leukopenia, thrombocytopenia, myelosuppression, radiation esophagitis, radiation pneumonitis, and postoperative complications. 5) Other efficacy-related and safety indicators reported in the study.

(4) S: RCTs were included, regardless of blinding status.

#### 2.3.2 Exclusion criteria

1. P: Patients who have not been diagnosed with EC, or patients whose cancer cells have spread from other parts of the body to esophagus;
2. I/C: Interventions involving immunomodulators other than thymosin (e.g., interferon, interleukin-2, etc.), or inconsistencies in the baseline treatment protocols between the experimental and control groups;
3. O: Studies with incomplete data extraction and duplicated publications;
4. S: Non-RCT, including reviews, case reports, animal studies, or conference abstracts.

### 2.4 Study selection and data extraction

Retrieved literature was imported into EndNote 21.0. After manual duplication checks, 2 researchers (R.Z. and C.Z.) independently screened the studies. They first excluded obviously irrelevant studies by reviewing titles and abstracts, then downloaded and critically read the full texts of the remaining studies to determine final inclusion. Any disagreements during this process were resolved through discussion and negotiation involving a third researcher (T.S.).

Using a pre-designed Excel data extraction table, 2 researchers (T.S. and X.S.) independently extracted the following information:

1. Study basic information: First author, publication year, country, sample size, patient baseline characteristics (age, gender, tumor stage, treatment regimen, etc.);
2. Intervention details: Specific thymosin type, dosage, frequency, treatment course, and detailed regimen of conventional antitumor therapy;
3. Outcome measure data: All relevant efficacy and safety outcome data reported in the study;
4. Methodological characteristics: Random sequence generation method, allocation concealment, blinding implementation, and dropout/loss to follow-up rates.

### 2.5 Risk of bias assessment

Using the Cochrane Collaboration’s recommended risk of bias assessment tool for RCTs (RoB 2.0) (29), each included study was evaluated across 5 domains: randomization process, deviation from the intended intervention, missing outcome data, outcome measurement, and selective reporting of results. Each domain was rated as “low risk,” “high risk,” or “some concerns.” 2 assessors (S.T. and R.Z.) conducted independent evaluations and cross-checked results. Disagreements were resolved through arbitration by a third researcher (Ya.Z.).

### 2.6 Statistical analysis

Meta-analyses were conducted using Review Manager (version 5.4). For dichotomous variables, risk ratios (RR) and their 95% confidence intervals (CI) were reported. For continuous variables, mean differences (MD) or standardized mean differences (SMD) and their 95% CIs were reported. For time-to-event variables (OS, PFS), if studies reported hazard ratios (HR) with 95% CIs, these were directly pooled; otherwise, data extraction from survival curves was attempted. Heterogeneity among studies was assessed using the Q-test and I² statistic. If P ≥ 0.10 and I² ≤ 50%, heterogeneity was considered acceptable, and a fixed-effect model was used. If P < 0.10 or I² > 50%, indicating significant heterogeneity, a random-effects model was employed, and sources of heterogeneity were explored (30).

### 2.7 Subgroup analysis and sensitivity analysis

To explore heterogeneity sources and efficacy differences across clinical settings, we conducted meta-regression analyses and performed the following pre-specified subgroup analyses: (1) thymosin type; (2) control group treatment regimen; (3) elderly patients (≥ 60 years) vs. non-elderly patients (< 60 years). Additionally, sensitivity analysis was performed using STATA software (version 18.0) by sequentially excluding individual studies to assess the robustness of pooled results (31).

### 2.8 Quality of evidence

The quality of evidence for each outcome was assessed using GRADEpro (https://www.gradepro.org/). Evidence quality was downgraded or upgraded based on 5 factors: risk of bias, inconsistency, indirectness, imprecision, and publication bias. Evidence quality was ultimately categorized into four levels: “high,” “moderate,” “low,” and “very low” (32). 2 researchers, T.S. and C.Z., independently performed the GRADE assessments.

### 2.9 Trial sequential analysis

To prevent type I errors (false positives) arising from repeated testing in cumulative meta-analyses, we employed TSA using TSA software v.0.9.5.10 Beta (Copenhagen Trial Unit, Copenhagen, Denmark) for selected outcome measures. A pre-specified Type I error α = 5% (two-tailed) and power (1-β) = 80% were set, and the required information size was calculated based on existing study data. By constructing a TSA curve, we determined whether the current cumulative evidence had crossed the required non-effect/effect boundary or whether further studies were still needed (33, 34).

### 2.10 Publication bias

To assess the impact of potential publication bias, we employed a combination of qualitative and quantitative methods. For each outcome comprising 10 or more studies, we first conducted a visual qualitative assessment using funnel plots, followed by quantitative evaluation via Egger’s test. Where publication bias was found to be significant, we further employed “trim-and-fill” method to quantitatively estimate the effect of publication bias on the results.

## 3 Results

### 3.1 Search results and study characteristics

**Figure 1:**
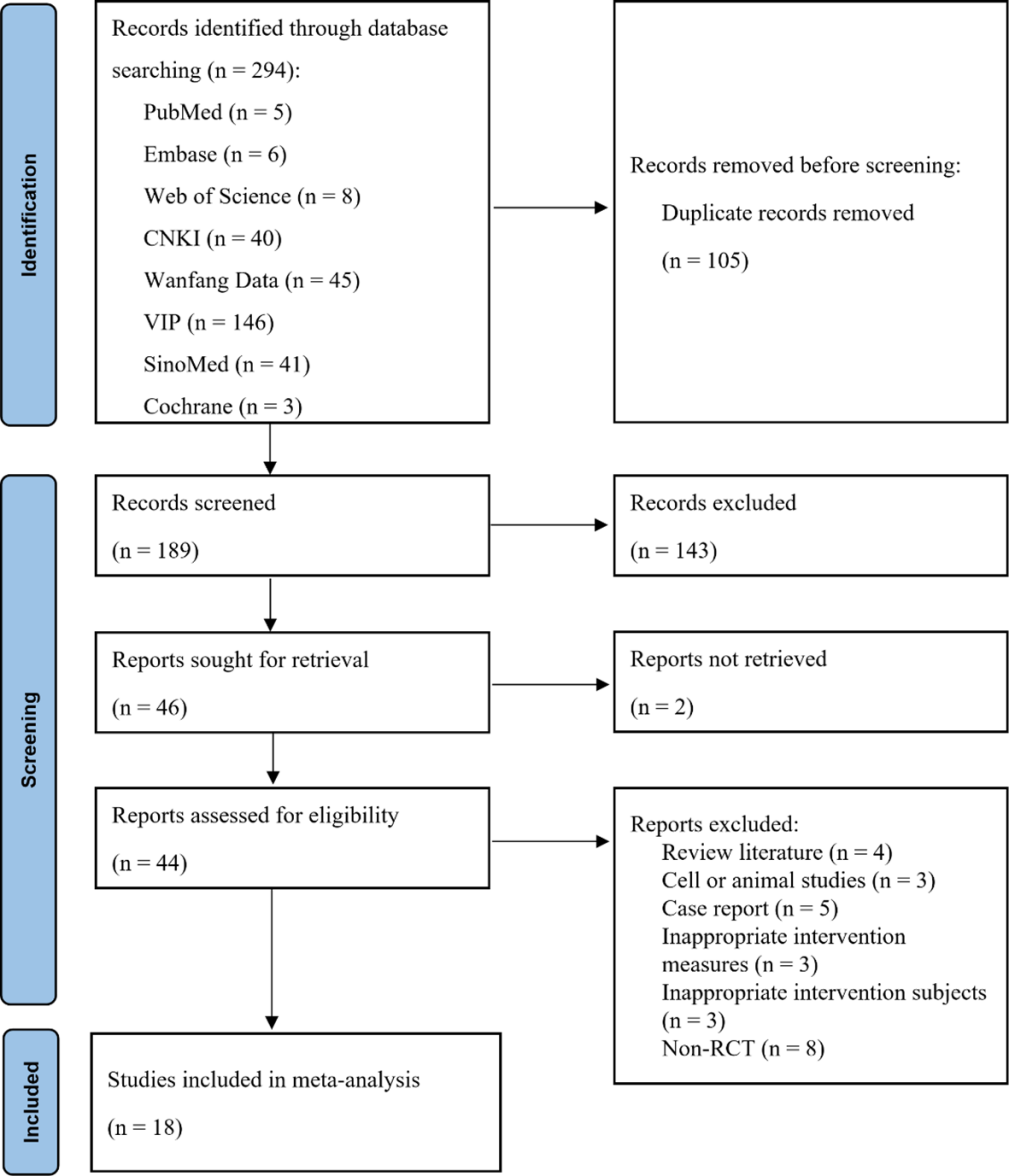
Flow diagram of the literature search.

Initial search identified 294 articles. After removing duplicates, 189 unique articles remained. Through preliminary screening of titles and abstracts, 143 articles that did not meet inclusion criteria were excluded. Subsequently, full-text review and assessment of the remaining 46 studies led to exclusion of 28 studies. Ultimately, 18 RCTs were included in meta-analysis (**Figure 1**). Regarding interventions in control group, 9 studies used radiotherapy alone, 3 studies used chemotherapy alone, 3 studies used concurrent chemoradiotherapy, and 3 studies used surgery combined with chemotherapy. Regarding interventions in treatment group, 13 studies used thymosin α1, 4 studies used thymosin injection, and 1 study used thymopentin. The baseline characteristics and detailed intervention protocols of included studies are summarized in **Table 1**.

**Table 1:** The characteristics of all included studies.

Author and reference	Sample size		Mean age (years)		Disease stage		Interventions		Radiotherapy (total dose)	Chemotherapy cycles (number)	Outcomes	Primary endpoint
	T	C	T	C	T	C	T	C				
Tang et al. (2011) (35)	29	28	M79.7	M78.9	No distant metastases		Thymosin α1	Radiotherapy	64–68 Gy	–	(3)(5)	1-, 3-year survival rate
Yang et al. (2012) (36)	24	24	67.8 ± 1.4	67.7 ± 1.3	–		Thymosin α1	Radiotherapy	64–68 Gy	–	(1)(2)(3)(5)	ORR and DCR
Zhang et al. (2012) (37)	34	33	53.48 ± 4.76		No distant metastases		Thymosin α1	Radiotherapy + Chemotherapy	60 Gy	PF	(1)(3)(4)(5)(9)	ORR and DCR
Su et al. (2013) (22)	46	42	61.46	59.86	II (21), III (59), IV (8)		Thymopentin	Radiotherapy + Chemotherapy	60–70 Gy	PF	(1)(2)(3)(4)(5)(8)(9)	ORR and DCR
Wu et al. (2013) (38)	23	23	60(55-67)		No distant metastases		Thymosin α1	Radiotherapy + Chemotherapy	60–70 Gy	PF	(1)(2)(9)	ORR and DCR
Zhu et al. (2013) (39)	35	35	< 70		No evidence of cancer metastasis		Thymosin Injection	Radiotherapy	64 Gy	–	(1)(2)(3)(9)	ORR and DCR
Lin et al. (2014) (40)	27	23	64.8 ± 2.1		Advanced		Thymosin α1	Chemotherapy	–	TC	(1)(2)(6)(7)(9)	CD4^+^%, CD4^+^/CD8^+^, and NK cell level
Suo et al. (2014) (41)	23	23	53(46-70)		Advanced		Thymosin Injection	Chemotherapy	–	PF	(1)(3)(5)(9)	ORR and DCR
Gao et al. (2015) (42)	35	29	66 ± 7		Resectable		Thymosin α1	Chemotherapy + Surgery	–	DP	(6)(7)(9)	CD4^+^%, CD4^+^/CD8^+^, and NK cell level
Zhang et al. (2015) (43)	30	30	61.4 ± 2.6	60.5 ± 2.3	–		Thymosin α1	Radiotherapy	60–70 Gy	–	(6)(7)(9)	CD4^+^%, CD4^+^/CD8^+^, and NK cell level
Geng et al. (2016) (44)	40	40	63.1 ± 4.9	62.8 ± 5.2	II (15), III (25)	II (18), III (22)	Thymosin α1	Chemotherapy + Surgery	–	DP	(6)(7)(9)(10)	CD4^+^%, CD4^+^/CD8^+^, and NK cell level
Wei et al. (2016) (45)	45	45	66.83 ± 6.26	66.25 ± 7.01	II (30), III (15)	II (28), III (17)	Thymosin α1	Radiotherapy	60 Gy	–	(1)(2)(6)	ORR and DCR
Lu et al. (2017) (46)	60	60	52.7 ± 6.1	52.1 ± 5.9	II (25), III (35)	II (29), III (34)	Thymosin α1	Radiotherapy	65–75 Gy	–	(1)(2)(3)(4)(5)(9)	ORR and DCR
Chen et al. (2018) (47)	23	23	63.1 ± 4.9	62.8 ± 5.2	II (9), III (14)	II (10), III (13)	Thymosin α1	Chemotherapy + Surgery	–	DP	(6)(7)(9)(10)	CD4^+^%, CD4^+^/CD8^+^, and NK cell level
Shan et al. (2020) (24)	50	50	53.25 ± 6.18	54.37 ± 6.24	–		Thymosin Injection	Radiotherapy	50 Gy	–	(1)(2)(6)(7)(9)	ORR and DCR
Li et al. (2020) (48)	30	30	61.0 ± 10.5	60.3 ± 11.3	II (14), III (16)	II (12), III (18)	Thymosin Injection	Radiotherapy	50 Gy	–	(1)(2)(6)(9)	ORR and DCR
Wang et al. (2020) (49)	30	30	51.5 ± 2.1	52.2 ± 3.1	II (21), III (9)	II (23), III (7)	Thymosin α1	Radiotherapy	60 Gy	–	(1)(2)(6)(9)	ORR and DCR
Xu et al. (2024) (23)	60	60	67.78 ± 1.19	68.12 ± 1.05	II (35), III (25)	II (33), III (27)	Thymosin α1	Chemotherapy	–	TP	(1)(2)(3)(6)(8)(9)	ORR and DCR

T, treatment group; C, control group. Outcomes, (1)ORR; (2)DCR; (3)1-year survival rate; (4)2-year survival rate; (5)3-year survival rate; (6)T lymphocyte subsets (CD3^+^%, CD4^+^%, CD8^+^%, and CD4^+^/CD8^+^); (7)NK cell level; (8)KPS improvement rate; (9)Incidence of treatment-related adverse effects; (10)Postoperative complications.

### 3.2 Risk of bias assessment

**Figure 2:**
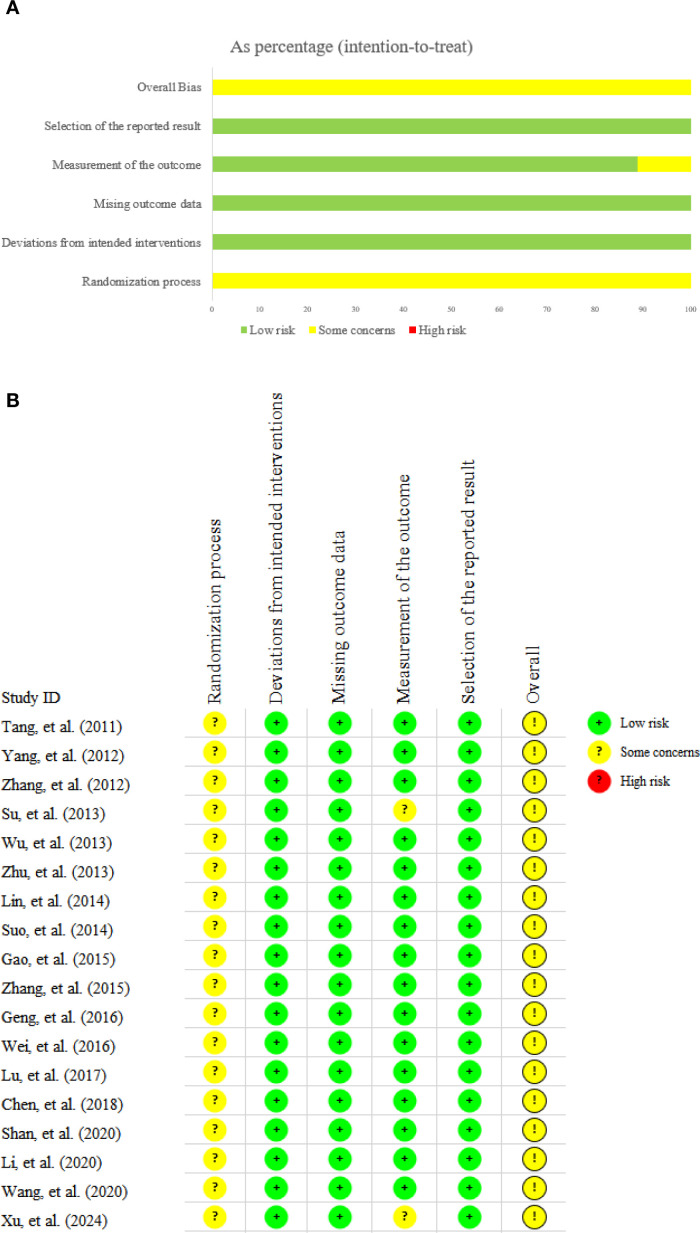
Risk of bias of included study. **(A)** risk of bias summary; **(B)** risk of bias graph.

Regarding methodological quality, none described specific implementation details for allocation concealment or blinding, resulting in a rating of “some concerns” for the “randomization process” dimension. For “outcome measurement,” 2 studies were also rated as “some concerns” due to potential subjective influences on the assessed outcome measures. However, all studies clearly defined primary outcome measures and provided complete data reporting. No substantial evidence of bias was identified in the dimensions of “deviation from the specified intervention,” “missing data,” and “selective reporting of results.” Consequently, the risk of bias in these areas was judged as “low risk.” Overall, RCTs included in this meta-analysis exhibited certain methodological limitations. Detailed results of bias risk assessment are summarized in **Figure 2**.

### 3.3 Primary outcomes

#### 3.3.1 ORR

**Figure 3:**
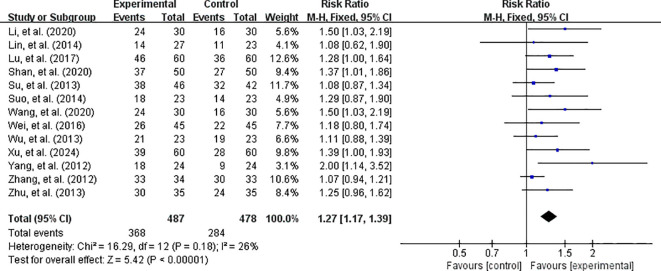
Forest plot of ORR.

13 RCTs (965 EC patients) reported ORR. Heterogeneity testing did not indicate significant heterogeneity (p = 0.18, I² = 26%), thus a fixed-effect model was used for pooled analysis. Meta-analysis revealed a higher ORR in the thymosin-combination therapy group compared to standard therapy (RR = 1.27, 95% CI: 1.17–1.39, p < 0.00001) (**Figure 3**). To further explore efficacy differences across clinical settings, subgroup analyses were conducted (see **Supplementary Material S3**).

Based on different thymosin preparations, patients were categorized into thymosin α1 group, thymosin injection group, and thymopentin group. Results showed that thymosin α1 group (RR = 1.27, 95% CI: 1.14–1.43, p < 0.0001) and thymosin injection group (RR = 1.35, 95% CI: 1.14–1.58, p = 0.0004) showed improved ORR compared to control group, with thymosin injection group exhibiting a more pronounced increase.

Based on different treatment regimens in control group, patients were categorized into radiotherapy-only, chemotherapy-only, and concurrent chemoradiotherapy groups. Results showed that compared with control group, radiotherapy-only group (RR = 1.37, 95% CI: 1.20–1.55, p < 0.00001) and chemotherapy-only group (RR = 1.30, 95% CI: 1.03–1.64, p = 0.03) showed significantly improved ORR, whereas no significant difference was observed in concurrent chemoradiotherapy group.

#### 3.3.2 DCR

**Figure 4:**
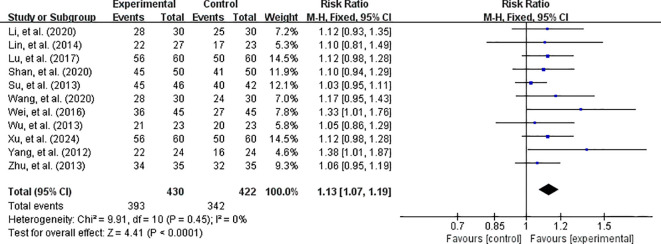
Forest plot of DCR.

11 RCTs (852 EC patients) reported DCR. No significant heterogeneity was detected between studies (p = 0.45, I² = 0%), thus a fixed-effect model was applied. Meta-analysis revealed that DCR was higher in thymosin-combination therapy group than in control group (RR = 1.13, 95% CI: 1.07–1.19, p < 0.0001) (**Figure 4**).

Subgroup analysis indicated (see **Supplementary Material S3**):

Thymosin α1 (RR = 1.16, 95% CI: 1.08–1.26, p < 0.0001) and thymosin injection (RR = 1.09, 95% CI: 1.00–1.20, p = 0.06) both improved DCR, with a more pronounced effect observed in thymosin α1 group.

Regarding different treatment regimens, combination therapy with thymosin showed a significant DCR benefit only when used with radiotherapy alone (RR = 1.16, 95% CI: 1.08–1.24, p < 0.0001), while no significant difference was observed in chemotherapy-only or concurrent chemoradiotherapy groups.

### 3.4 Secondary outcomes

#### 3.4.1 1/2/3-year survival rates

Since the vast majority of included RCTs reported survival rates at specific time points without providing survival curves, HR, and their 95% CI, we used RR to pool the 1-, 2-, and 3-year survival rates.

**Figure 5:**
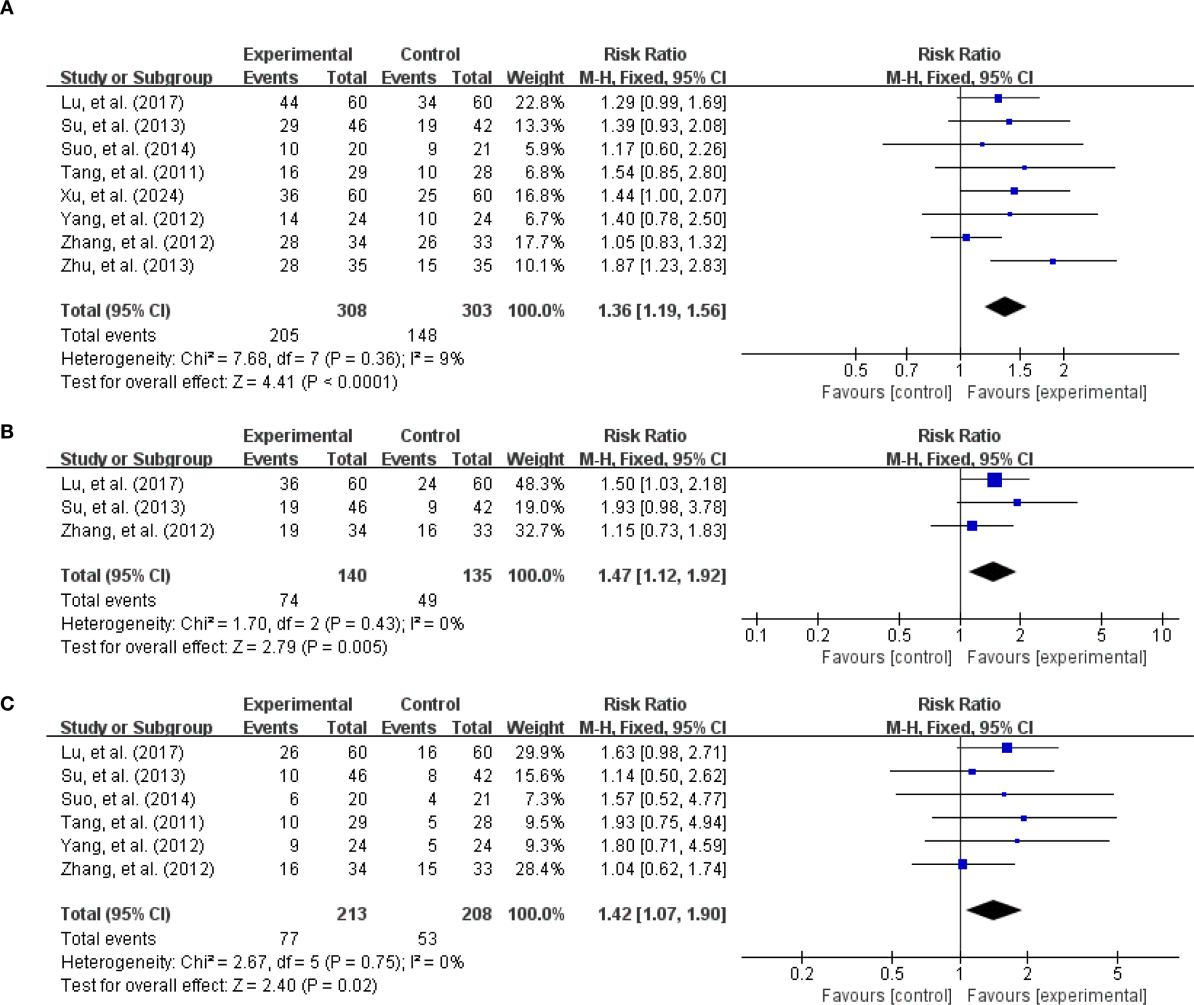
**(A)** Forest plot of 1-year survival rate. **(B)** Forest plot of 2-year survival rate. **(C)** Forest plot of 3-year survival rate. 1/2/3-year survival rates: **(A)** 1-year survival rate; **(B)** 2-year survival rate; **(C)** 3-year survival rate.

A total of 8, 3, and 6 RCTs reported 1-, 2-, and 3-year survival rates, involving 611, 275, and 421 patients, respectively. No significant heterogeneity was detected in any analysis (1-year survival rate: p = 0.36, I² = 9%; 2-year survival rate: p = 0.43, I² = 0%; 3-year survival rate: p = 0.75, I² = 0%), thus fixed-effect models were applied. Pooled analysis showed that combined thymosin therapy group demonstrated significantly improved 1-year survival rate (RR = 1.36, 95% CI: 1.19–1.56, p < 0.0001, **Figure 5A**), 2-year survival rate (RR = 1.47, 95% CI: 1.12–1.92, p = 0.005, **Figure 5B**), and 3-year survival rate (RR = 1.42, 95% CI: 1.07–1.90, p = 0.02, **Figure 5C**), which were significantly higher than those in control group (**Figure 5**).

#### 3.4.2 T lymphocyte subsets

**Figure 6:**
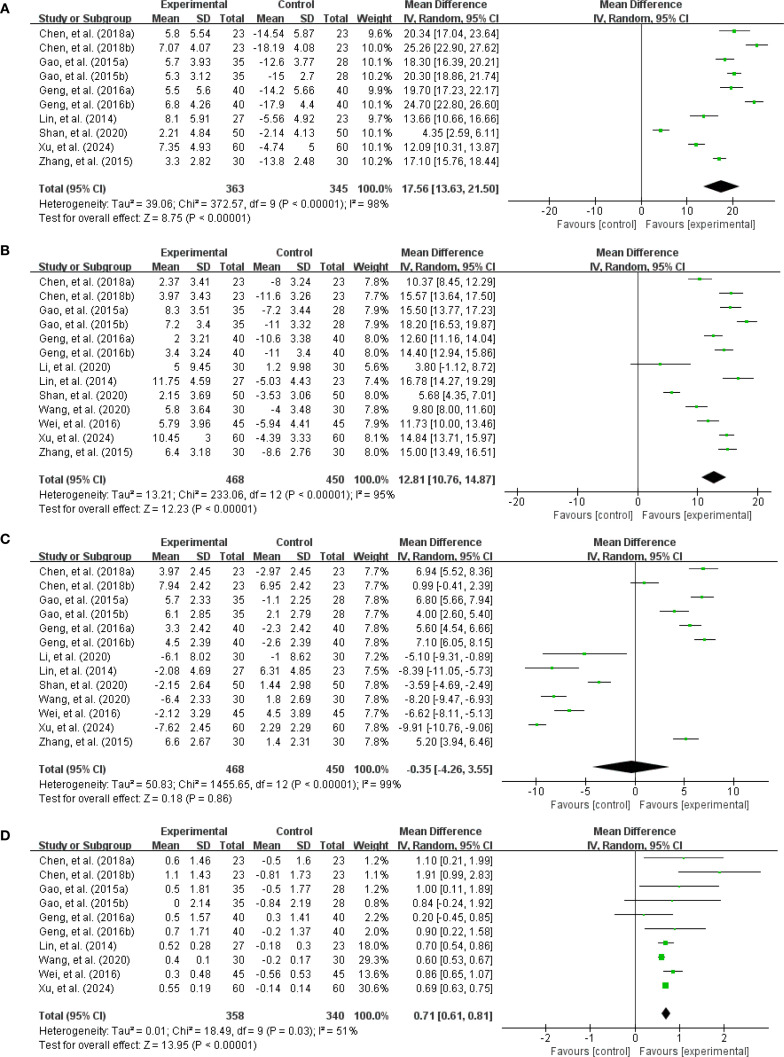
**(A)** Forest plot of CD3^+^%. **(B)** Forest plot of CD4^+^%. **(C)** Forest plot of CD8^+^%. **(D)** Forest plot of CD4^+^/CD8^+^ ratio. Forest plot of T lymphocyte subsets: **(A)** CD3^+^%; **(B)** CD4^+^%; **(C)** CD8^+^%; **(D)** CD4^+^/CD8^+^ ratio.

Analysis of T lymphocyte subsets included: CD3^+^% (10 RCTs, 708 patients), CD4^+^% (13 RCTs, 918 patients), CD8^+^% (13 RCTs, 918 patients), and CD4^+^/CD8^+^ ratio (10 RCTs, 698 patients). Pooled analysis revealed that compared with control group, treatment group with thymosin showed a significant increase in CD3^+^% (MD = 17.56, 95% CI: 13.63 - 21.50, p < 0.00001, **Figure 6A**), CD4^+^% (MD = 12.81, 95% CI: 10.76 - 14.87, p < 0.00001, **Figure 6B**), and CD4^+^/CD8^+^ ratio (MD = 0.71, 95% CI: 0.61 - 0.81, p < 0.00001, **Figure 6D**). However, no significant effect was observed on CD8^+^% (MD = -0.35, 95% CI: -4.26–3.55, p = 0.86, **Figure 6C**).

Given extremely high heterogeneity among studies, we conducted meta-regression and subgroup analyses based on thymosin type, combination therapy, and patient age; however, these did not fully account for sources of heterogeneity. Detailed results are presented in **Supplementary Material S3**.

#### 3.4.3 NK cell level

**Figure 7:**
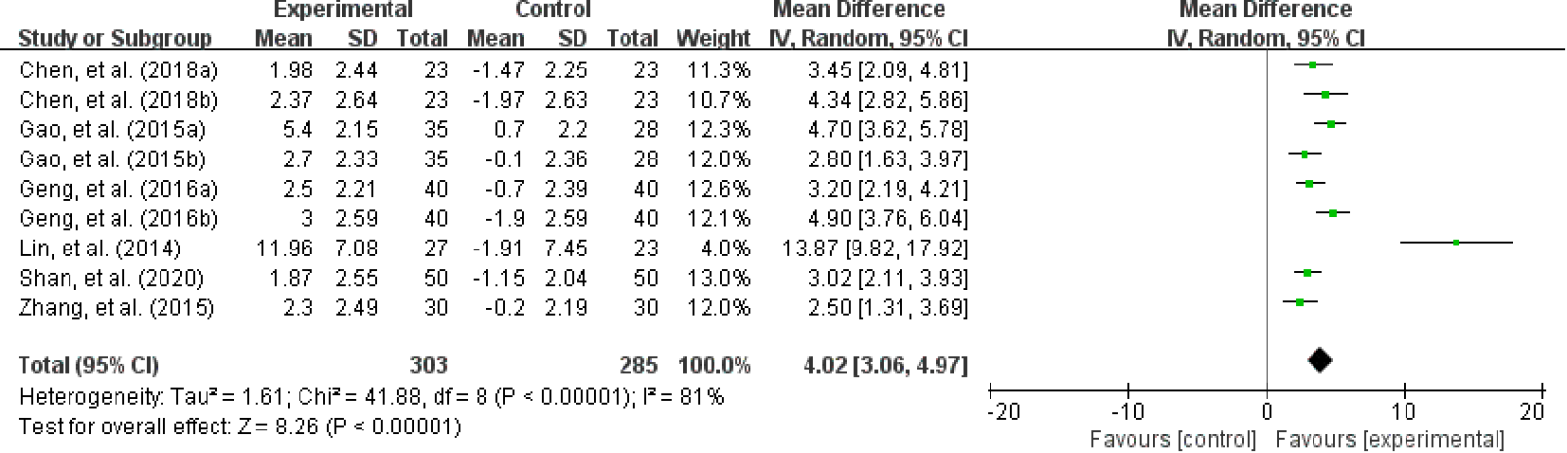
Forest plot of NK cell level.

9 RCTs (588 EC patients) reported NK cell level. Due to high heterogeneity between studies (p < 0.00001, I² = 81%), a random-effects model was employed. Results showed significantly higher NK cell level in thymosin-combination therapy group compared to control group (MD = 4.02, 95% CI: 3.06–4.97, p < 0.00001, **Figure 7**). Exploratory analyses addressing this high heterogeneity are detailed in **Supplementary Material S3**.

#### 3.4.4 KPS improvement rate

**Figure 8:**
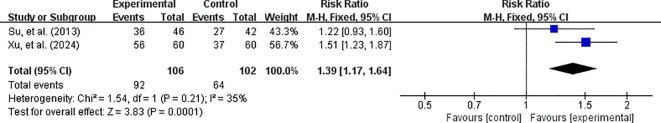
Forest plot of KPS improvement rate.

2 RCTs (208 EC patients) reported KPS improvement rates. Inter-study heterogeneity was not significant (p = 0.21, I² = 35%) using a fixed-effect model. Pooled analysis indicated that KPS improvement rate in treatment group was significantly higher than that in control group (RR = 1.39, 95% CI: 1.17–1.64, p = 0.0001) (**Figure 8**). However, given the limited number of studies, this conclusion should be interpreted with caution.

#### 3.4.5 Incidence of treatment-related adverse effects

##### 3.4.5.1 Gastrointestinal reactions

**Figure 9:**
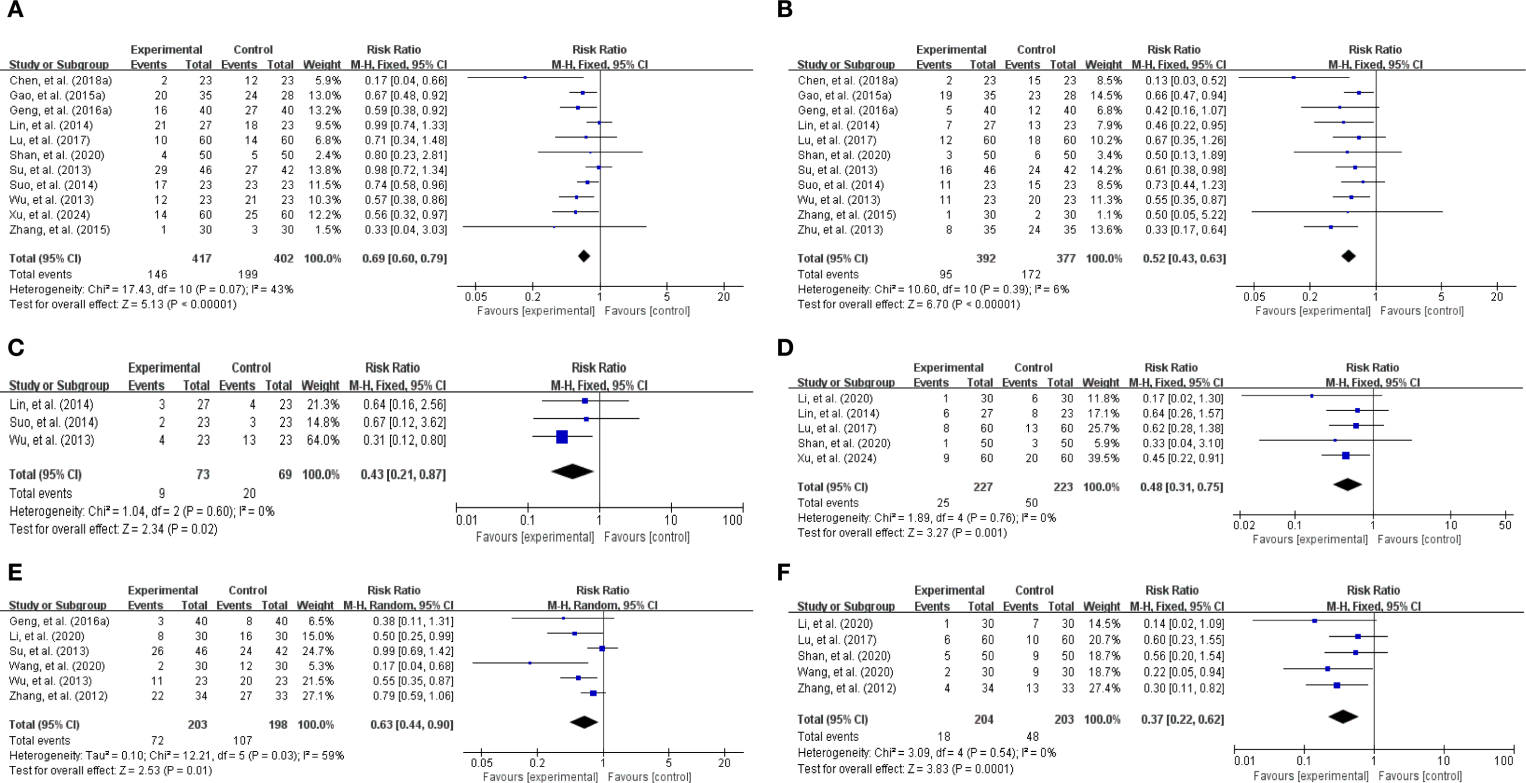
**(A)** Forest plot of gastrointestinal reactions. **(B)** Forest plot of leukopenia. **(C)** Forest plot of thrombocytopenia. **(D)** Forest plot of myelosuppression. **(E)** Forest plot of radiation esophagitis. **(F)** Forest plot of radiation pneumonitis. Forest plot of incidence of treatment-related adverse effects: **(A)** gastrointestinal reactions; **(B)** leukopenia; **(C)** thrombocytopenia; **(D)** myelosuppression; **(E)** radiation esophagitis; **(F)** radiation pneumonitis.

11 RCTs (819 EC patients) reported incidence of gastrointestinal reactions. Heterogeneity testing revealed no significant heterogeneity (p = 0.07, I² = 43%), thus allowing for use of a fixed-effect model to pool effect sizes. Results indicate that incidence of gastrointestinal reactions was significantly lower in thymosin-combination therapy group (RR = 0.69, 95% CI: 0.60–0.79, p < 0.00001) (**Figure 9A**).

##### 3.4.5.2 Leukopenia, thrombocytopenia, and myelosuppression

11, 3, and 5 RCTs reported incidence rates of leukopenia, thrombocytopenia, and myelosuppression, respectively, involving 769, 142, and 450 EC patients. No significant heterogeneity was detected among studies (leukopenia: p = 0.39, I² = 6%; thrombocytopenia: p = 0.60, I² = 0%; myelosuppression: p = 0.76, I² = 0%). Therefore, a fixed-effect model was applied. Meta-analysis indicated that thymosin-combination therapy group significantly reduced leukopenia (RR = 0.52, 95% CI: 0.43–0.63, p < 0.00001, **Figure 9B**), thrombocytopenia (RR = 0.43, 95% CI: 0.21–0.87, p = 0.02, **Figure 9C**), and myelosuppression (RR = 0.48, 95% CI: 0.31–0.75, p = 0.001, **Figure 9D**).

##### 3.4.5.3 Radiation esophagitis and radiation pneumonitis

6 RCTs (401 EC patients) reported incidence of radiation esophagitis. Heterogeneity testing revealed moderate heterogeneity (p = 0.03, I² = 59%), thus a random-effects model was applied. Results indicated a lower incidence of radiation esophagitis in treatment group (RR = 0.63, 95% CI: 0.44–0.90, p = 0.01, **Figure 9E**). 5 RCTs (407 EC patients) reported incidence of radiation pneumonitis. No significant heterogeneity existed between studies (p = 0.54, I² = 0%), thus a fixed-effect model was applied. Results demonstrated a similarly lower incidence of radiation pneumonitis in treatment group (RR = 0.37, 95% CI: 0.22–0.62, p = 0.0001, **Figure 9F**).

##### 3.4.5.4 Postoperative adverse reactions

**Figure 10:**
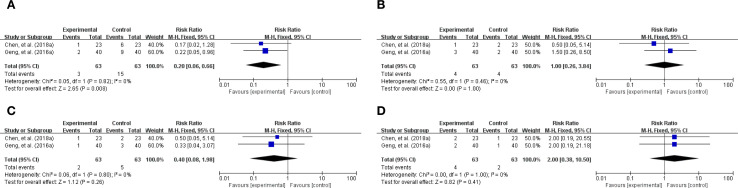
**(A)** Forest plot of postoperative pulmonary infection. **(B)** Forest plot of postoperative atelectasis. **(C)** Forest plot of postoperative anastomotic fistula. **(D)** Forest plot of postoperative arrhythmia. Forest plot of postoperative complications: **(A)** postoperative pulmonary infection; **(B)** postoperative atelectasis; **(C)** postoperative anastomotic fistula; **(D)** postoperative arrhythmia.

2 RCTs (126 EC patients) reported postoperative adverse reaction rates (including postoperative pulmonary infection, postoperative atelectasis, postoperative anastomotic fistula, and postoperative arrhythmia). No significant heterogeneity existed between studies (postoperative pulmonary infection: p = 0.82, I² = 0%; postoperative atelectasis: p = 0.46, I² = 0%; postoperative anastomotic fistula: p = 0.80, I² = 0%; postoperative arrhythmia: p = 1.00, I² = 0%). Therefore, a fixed-effect model was applied. Results indicated that combined thymosin therapy was associated only with a significant reduction in risk of postoperative pulmonary infection (RR = 0.20, 95% CI: 0.06–0.66, p = 0.008, **Figure 10A**), with no significant effect on other complications (**Figure 10**). However, given the limited number of studies, this conclusion should be interpreted with caution.

### 3.5 Sensitivity analysis

**Figure 11:**
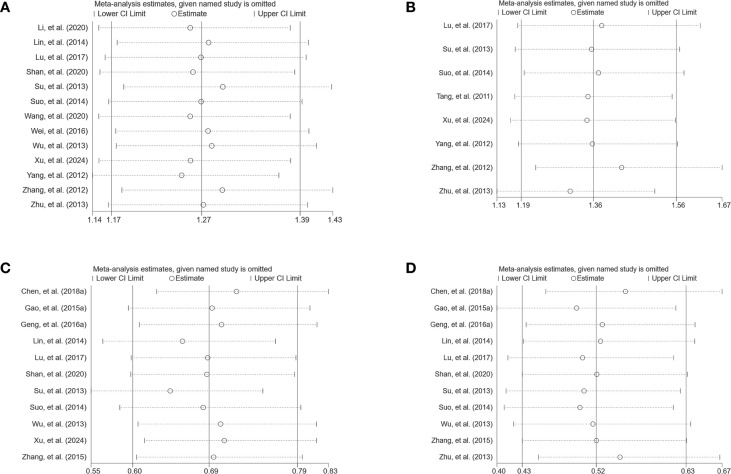
Sensitivity analyses of primary outcomes: **(A)** ORR; **(B)** 1-year survival rate; **(C)** gastrointestinal reactions; **(D)** leukopenia.

Sensitivity analysis using a stepwise exclusion method indicated that pooled effect size remained essentially unchanged for outcomes including ORR, DCR, 1-year survival rate, CD3^+^%, CD4^+^%, CD4^+^/CD8^+^ ratio, NK cell level, gastrointestinal reactions, leukopenia, myelosuppression, radiation esophagitis, and radiation pneumonitis, suggesting stable results. However, pooled results for 2-year survival rate, 3-year survival rate, and thrombocytopenia showed changes in the sensitivity analysis, indicating limited stability for these conclusions. Key findings from the sensitivity analysis (using ORR, 1-year survival rate, gastrointestinal reactions, and leukopenia as examples) are presented in **Figure 11**, with full results available in **Supplementary Material S3**.

### 3.6 Quality of evidence

GRADE system was applied to assess quality of evidence. Evidence grades for ORR, DCR, and 1/2/3-year survival rates were rated as “low”; Evidence grade for leukopenia was rated as “moderate.” Evidence grades for most other outcomes were “low” or “very low,” primarily downgraded due to methodological limitations (e.g., lack of blinding and allocation concealment reporting), substantial statistical heterogeneity, overly wide confidence intervals, and insufficient study numbers. Detailed assessment results see **Supplementary Material S4**.

### 3.7 TSA

**Figure 12:**
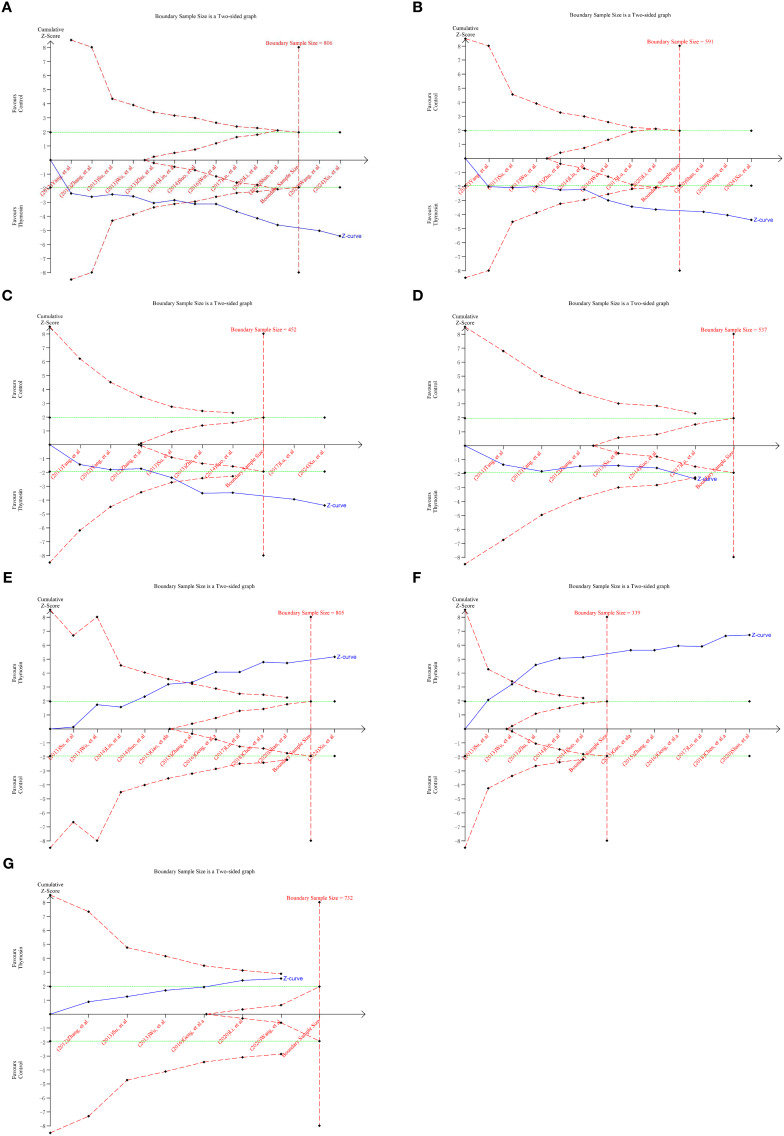
Outcomes of TSA. **(A)** ORR; **(B)** DCR; **(C)** 1-year survival rate; **(D)** 3-year survival rate; **(E)** gastrointestinal reactions; **(F)** leukopenia; **(G)** radiation esophagitis.

TSA results (**Figure 12**) indicate that for outcomes including ORR, DCR, 1-year survival rate, gastrointestinal reactions, and leukopenia, cumulative Z-curves transcends both conventional boundary and TSA boundary. Furthermore, sample size has reached the level required to detect predefined effect size, indicating that conclusions regarding efficacy are statistically convergent; however, this does not reduce risk of bias arising from low quality of individual studies.

However, for 3-year survival rate, while cumulative Z-curve crossed both conventional and TSA boundaries, sample size has not yet reached required information level. For radiation esophagitis, although cumulative Z-curve crossed conventional boundary, it did not cross TSA boundary, suggesting a potential false positive result. Further studies are still needed to obtain more accurate conclusions.

### 3.8 Publication bias

**Figure 13:**
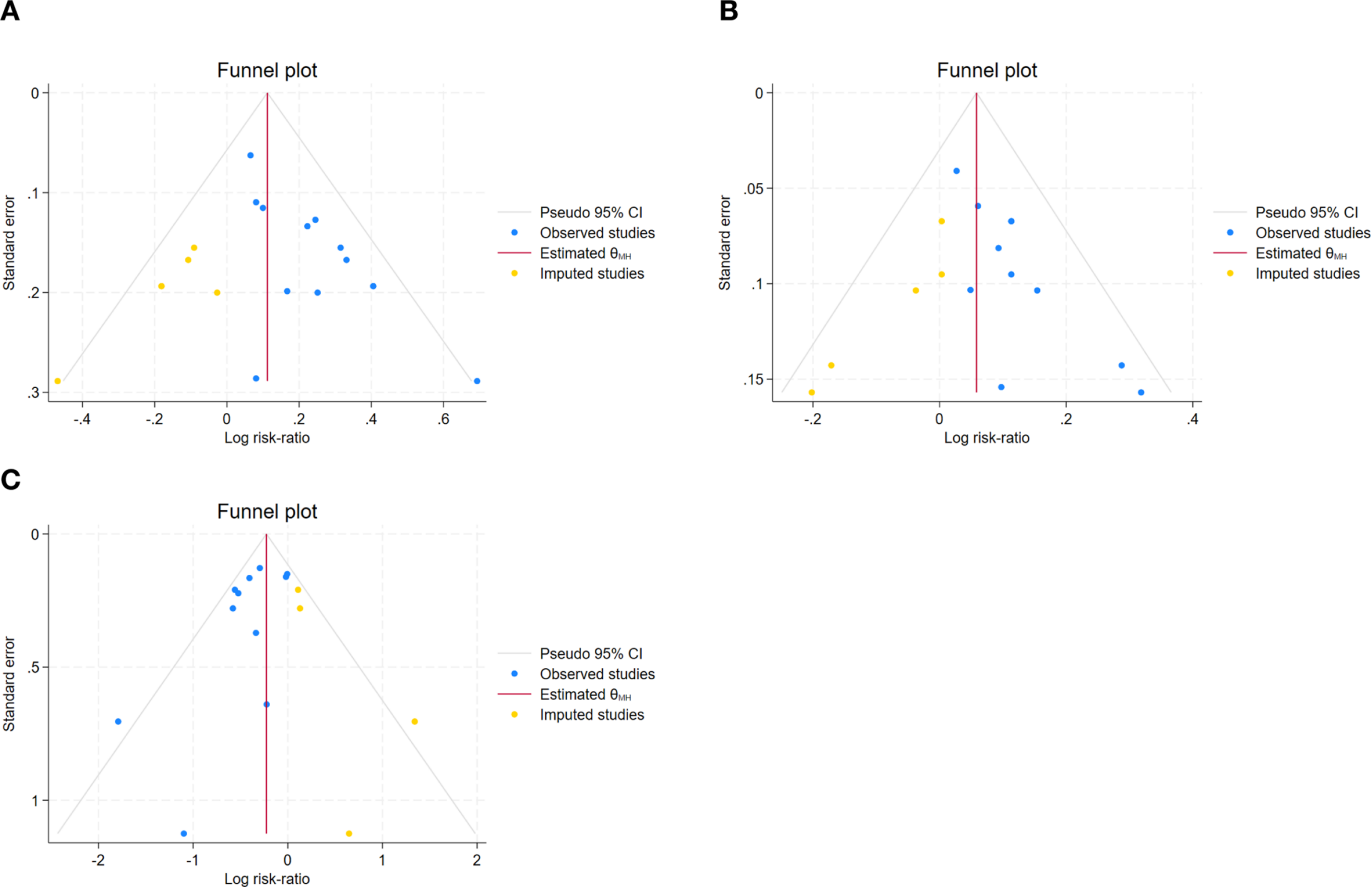
After “trim and fill” method of selected outcomes: **(A)** ORR; **(B)** DCR; **(C)** gastrointestinal reactions.

Publication bias assessment was conducted for 8 outcome measures (ORR, DCR, CD3^+^%, CD4^+^%, CD4^+^/CD8^+^ ratio, gastrointestinal reactions, and leukopenia). Their funnel plots are presented in **Supplementary Material S3**, with Egger’s test results shown in **Table 2**. To assess the impact of publication bias on outcomes, “trim-and-fill” method were further applied to selected results (ORR, DCR, and gastrointestinal reactions). Findings indicated limited bias influence: ORR (observed: RR = 1.185, 95% CI: 1.102–1.274; observed + imputed: RR = 1.119, 95% CI: 1.048–1.194, **Figure 13A**); DCR (observed: RR = 1.088, 95% CI: 1.040–1.138; observed + imputed: RR = 1.060, 95% CI: 1.019–1.103, **Figure 13B**); gastrointestinal reactions (observed: RR = 0.748, 95% CI: 0.661–0.847; observed + imputed: RR = 0.798, 95% CI: 0.711–0.896, **Figure 13C**).

**Table 2:** Results of Egger’s test.

Outcomes	Egger’ test
ORR	P = 0.0060
DCR	P = 0.0296
CD3^+^%	P = 0.6583
CD4^+^%	P = 0.1165
CD4^+^/CD8^+^ radio	P = 0.0513
Gastrointestinal reactions	P = 0.0286
Leukopenia	P = 0. 0652

## 4 Discussion

This study represents the first systematic review and meta-analysis to comprehensively evaluate efficacy and safety of thymosin combined with anticancer therapy for EC patients. Through a pooled analysis of 18 RCTs involving 1,272 EC patients, we found that combining thymosin with standard anticancer therapy may not only improve short-term tumor response rates (ORR and DCR) and 1- to 3-year survival rates but also enhance patients’ immune function and reduced risk of multiple treatment-related adverse events. These findings provide the latest evidence-based support for thymosin’s immunomodulatory value and clinical benefits in the comprehensive management of EC. However, given the limited quality of included studies and lack of reports on adverse effects associated with thymosin preparations themselves, these conclusions still require further validation through high-quality, prospective RCTs.

### 4.1 Principal findings

One of the most significant findings of this study is the positive regulatory effect of thymosin on the patients’ immune systems. Analysis revealed that combination therapy may increase CD3^+^% and CD4^+^% in peripheral blood, elevate the CD4^+^/CD8^+^ ratio, and enhance NK cell level. This outcome aligns closely with the known biological functions of thymosin: Thymosin α1 promotes the differentiation and maturation of T cell precursors, enhances Th1-type immune responses, and stimulates dendritic cell function (50, 51); while thymopentin specifically induces T cell differentiation and regulates cytokine production (52). EC patients, particularly those undergoing chemoradiotherapy, frequently experience severe lymphopenia and immune dysfunction. This is closely associated with increased infection risk, poor treatment tolerance, and adverse prognosis (53, 54). Our study suggests that thymosin may create conditions for restoring antitumor immune surveillance by reversing this treatment-induced immunosuppression.

In terms of clinical efficacy, thymosin combination therapy demonstrated a certain synergistic effect. The increases in ORR and DCR suggest thymosin may enhance the antitumor effects of conventional therapies. Its potential mechanisms extend beyond systemic immune restoration: Previous studies on mechanisms have shown that thymosin modulates immune cell infiltration in the tumor microenvironment, enhances effector cell killing activity against tumors, and may induce tumor cell apoptosis by blocking relevant tumor suppressor genes (55, 56). The survival benefit analysis is particularly encouraging. The concurrent improvement in 1-, 2-, and 3-year survival rates, though requiring higher-level evidence, strongly suggests that enhanced immune function may correlate with long-term survival benefits. This aligns with trends observed in other cancers such as lung and liver cancer, where immune status emerges as an independent prognostic factor for tumor patients (57, 58).

Regarding safety, studies included in this meta-analysis consistently reported that thymosin combination therapy was well tolerated and significantly reduced the risk of multiple adverse events associated with conventional treatment. However, it is important to note that this research focused on common toxicities related to radiotherapy and chemotherapy, with no systematic reporting on adverse reactions specific to thymosin preparations themselves (such as allergic reactions). This constitutes a gap in safety knowledge. Thymosin injection, as a mixture of biopeptides extracted from animal thymus, has a complex composition and relatively low purity, posing a potential risk of sensitization. There have been isolated case reports of allergic reactions in clinical use (59). Studies have shown that allergic reactions caused by thymosin injection may be related to cross-sensitivity to heterologous proteins (such as bovine serum albumin) (59). In contrast, Thymosin α1 (Thymalfasin) is a chemically synthesized single peptide consisting of 28 amino acids. Its composition is well-defined, its purity is higher, and it theoretically offers superior safety (57). Multiple clinical studies have also confirmed that Thymosin α1 exhibits favorable safety profiles (60–62). Thymopentin is a synthetic peptide composed of five amino acids—arginine, lysine, aspartic acid, valine, and tyrosine—and represents the biologically active fragment of thymopoietin II that possesses all physiological functions. With a well-defined structure and high purity, it does not require a skin test, and its safety profile falls between that of thymosin α1 and thymosin injection. In addition to allergic reactions, thymosin preparations may cause other rare adverse reactions, including nausea, fever, dizziness, chest tightness, weakness, and drowsiness. These symptoms are typically mild and tolerable. During long-term treatment, some patients may experience transient elevations in liver enzymes, which may be related to drug-induced immunomodulatory effects rather than direct hepatotoxicity (60). Therefore, while current evidence supports the safety of thymosin combination therapy, vigilance regarding the allergic risk of thymosin preparations—particularly thymosin injection—remains essential in future clinical practice and research. More precise documentation and reporting of drug-related adverse events are strongly encouraged. Future development efforts should focus on further optimizing formulation processes and clarifying active components to enhance efficacy while minimizing potential risks.

### 4.2 Pharmacological differences among thymosin preparations and their clinical implications

The results of subgroup analysis provide guidance for the differentiated application of thymosin. Both Thymosin-α1 and Thymosin injection increased ORR and DCR; however, there may be underlying differences between them, which are likely closely related to the pharmacological characteristics of different Thymosin formulations. Thymosin α1 (Thymosin-α1) meets the five criteria established by the WHO for immunomodulators. Its mechanism of action exhibits multi-target characteristics: (1) It activates the TLR7/SHIP1 axis, reprogramming M2 macrophages into M1 macrophages and enhances the antitumor immune response (54); (2) Binding to phosphatidylserine on the surface of apoptotic tumor cells, promoting the uptake of apoptotic bodies by dendritic cells, and upregulating the expression of MHC class II molecules and the co-stimulatory molecule CD86, thereby enhancing tumor antigen presentation capacity (63); (3) It modulates the Th1/Th2 cytokine balance, promoting the production of Th1 cytokines such as IL-2 and IFN-γ, and enhancing the tumor infiltration and cytotoxic activity of CD8^+^ T cells (50, 64); (4) It reduces the proliferation of regulatory T cells in the tumor microenvironment, thereby lifting immune suppression (64). These multi-level immune regulatory mechanisms collectively explain why thymosin α1 offers superior advantages in the stable control of the disease.

Thymosin injection contains multiple active components, including thymosin α1 and thymopoietin, but it also contains immunogenic macromolecular proteins. Due to differences in manufacturing processes and standards, there is significant heterogeneity in product quality and clinical efficacy among different manufacturers. While the complexity of its composition may confer multi-target immune regulatory effects, it also results in an unclear mechanism of action and poor stability of therapeutic efficacy. Thymopentin has a half-life of only 30 seconds, requiring frequent injections to maintain therapeutic concentrations, which may compromise its efficacy stability in long-term disease control. In the subgroup analysis of this study, thymosin pentapeptide was used in only one study, which is insufficient to draw definitive conclusions regarding its efficacy.

Subgroup analysis also revealed that the benefits of thymosin in improving ORR and DCR were most evident and significant when combined with radiotherapy alone or chemotherapy alone; however, in the subgroup receiving concurrent chemoradiotherapy, short-term efficacy did not reach statistical significance. This may be related to the stronger immunosuppressive effects of concurrent chemoradiotherapy. The combination of radiotherapy and chemotherapy can lead to more severe lymphocyte depletion and bone marrow suppression. In such cases, the “immune rescue” effect of thymosin may reach its efficacy ceiling, suggesting that the timing and dosage of thymosin administration need to be optimized in intensive combination regimens—such as through intensive treatment during radiotherapy-chemotherapy intervals or extended treatment cycles—to fully leverage its immunomodulatory effects.

In summary, thymosin α1 offers advantages in stabilizing disease control and improving long-term prognosis due to its well-defined structure, multi-target mechanism, and good safety profile; thymosin injection, however, requires cautious use due to its complex composition, inconsistent efficacy, and risk of allergic reactions; thymopentin, with its short half-life, may be more suitable for short-term immune support. Future research should explore optimized combination regimens and stratification strategies based on immune status.

### 4.3 Future directions for combination of thymosin preparations and modern immunotherapy in EC

With the breakthroughs achieved by immune checkpoint inhibitors in the treatment of EC, the combination of thymosin and PD-1/PD-L1 inhibitors has emerged as a key area of research. Thymosin α1 increases the number and activity of tumor-infiltrating lymphocytes by promoting T-cell maturation, enhancing antigen presentation, and remodeling the tumor microenvironment; Immune checkpoint inhibitors, in turn, lift tumor-induced immune suppression, and together they form a complete immune cycle of “priming-activation-effect.” Animal studies have confirmed that thymosin α1 can downregulate PD-L1 expression on tumor cells, thereby enhancing the efficacy of PD-1 inhibitors (65, 66). Furthermore, thymosin’s ability to mitigate immune damage caused by radiotherapy and chemotherapy can create favorable conditions for subsequent immunotherapy.

Future research should focus on: (1) Conducting RCTs of thymosin α1 in combination with PD-1 inhibitors to validate synergistic effects; (2) Exploring the feasibility of thymosin as an “immunopreparation” regimen prior to immunotherapy; (3) Evaluating the potential value of thymosin in the management of immune-related adverse events; (4) Establishing stratified treatment strategies based on immune status to identify optimal patient populations.

### 4.4 Limitations and future perspectives

Despite positive findings, this study has several limitations that warrant caution in interpreting conclusions.

First, the methodological quality of included studies was generally poor; none of trials mentioned allocation concealment or blinding. This lack of methodological rigor may lead to selection and performance biases, which could result in an overestimation of treatment efficacy. Furthermore, GRADE showed that certainty of evidence for most outcomes was rated as “very low” to “low.” Therefore, the pooled estimates and conclusions should be interpreted with caution. There is an urgent need for large-scale, methodologically rigorous RCTs—particularly those employing placebo-controlled and double-blind designs—to further validate these findings.

Second, significant heterogeneity was observed in analysis of certain outcome measures (such as NK cells). Although we conducted subgroup and meta regression analyses, underlying clinical and methodological heterogeneity—such as differences in thymosin dosage, treatment duration, cancer histology and stage, and patients’ baseline immune status—may not have been fully accounted for. Therefore, caution should be exercised when interpreting these results. Future studies should further investigate differences in the efficacy and safety of different thymosin preparations (particularly thymosin α1 and thymosin injection) in specific clinical settings and establish risk monitoring protocols.

Furthermore, all included studies were conducted in China, which may limit the generalizability of these findings to populations with different genetic backgrounds, treatment regimens, or healthcare systems. Additionally, Egger’s test indicated the presence of publication bias (for ORR, DCR, and gastrointestinal reactions), although results did not change significantly after applying “trim-and-fill” method. Therefore, caution should be exercised when interpreting the results, and future multicenter international studies are needed to validate applicability of thymosin combination therapy in different settings.

Finally, the lack of long-term survival data (such as 5-year overall survival or 5-year progression-free survival) and comparative data against current standard immunotherapy regimens limits the comprehensiveness of the conclusions. Although some studies reported 1-, 2-, and 3-year survival rates, since they did not provide specific survival curves, HR, and their 95% CI, we were limited to using RR for time-to-event analysis, which may have introduced bias in effect estimates. Given the clinical importance of long-term survival data for EC, future RCTs should extend follow-up period and report long-term survival outcomes to assess sustained survival benefits. At the same time, the synergistic potential and optimal regimens of combining thymosin with new therapies such as ICIs should be actively explored.

## 5 Conclusion

In summary, this meta-analysis suggests that combining thymosin with anticancer therapy may help enhance immune function in EC patients. It may also improve tumor response rates and short-term survival while reducing the risk of treatment-related adverse events. Although existing evidence supports thymosin’s positive value in EC treatment, further validation through rigorously designed prospective studies is warranted due to limitations in methodological quality of included RCTs and the presence of publication bias. This study provides new data supporting clinical application of thymosin and points way toward further exploration of its mechanism of action.

## Data Availability

Datasets are available on request: The raw data supporting the conclusions of this article will be made available by the authors, without undue reservation.
