# Supplementary material for: Efficacy and safety of thymosin combined with anticancer therapy for esophageal cancer: a systematic review and meta-analysis of randomized controlled trials
Source: Front Immunol. 2026 May 29;17:1812375. doi: 10.3389/fimmu.2026.1812375 (PMC13260294; doi:10.3389/fimmu.2026.1812375)
Supplement: Supplementary file 1 [file Table1.docx]

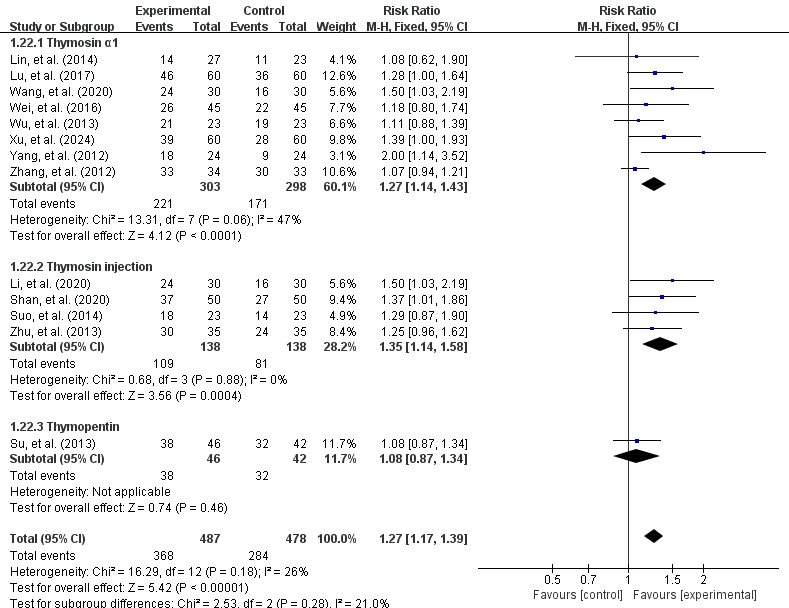


**Figure S1 (a):** Subgroup analysis of ORR (Thymosin type).


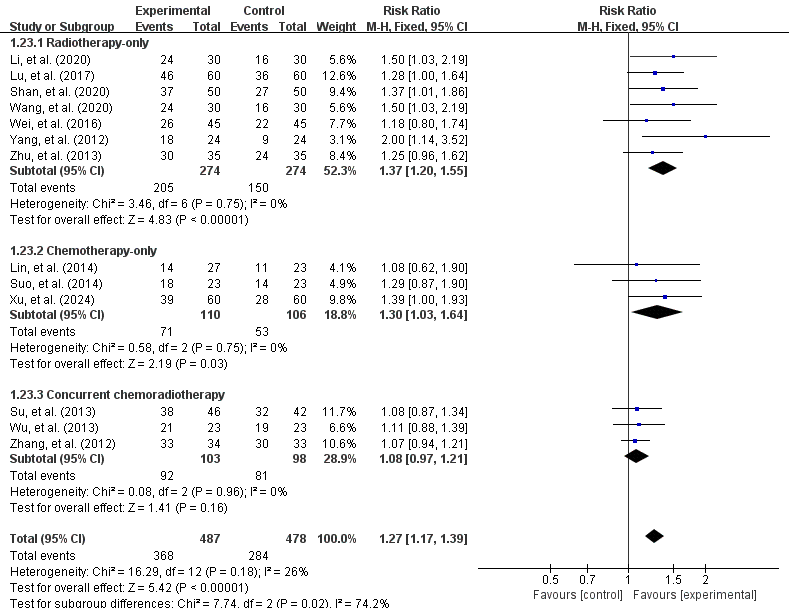


**Figure S1 (b):** Subgroup analysis of ORR (Control group treatment regimen).


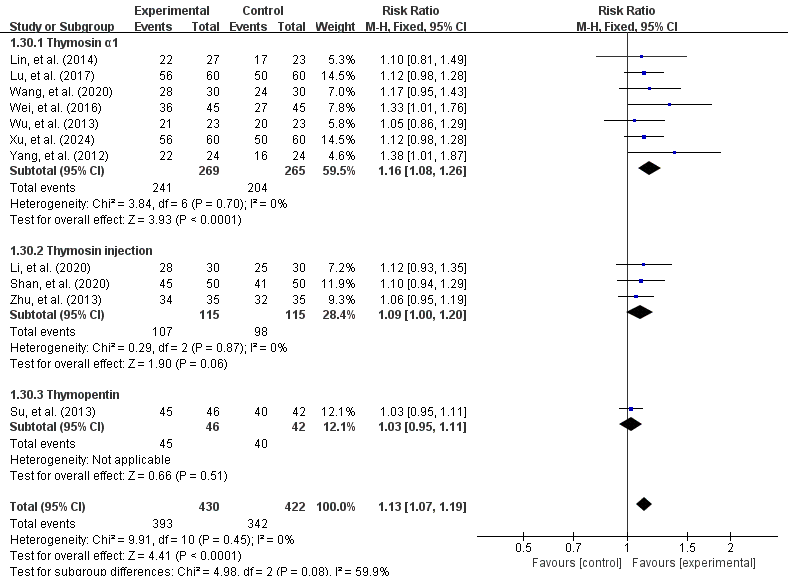


**Figure S1 (c):** Subgroup analysis of DCR (Thymosin type).


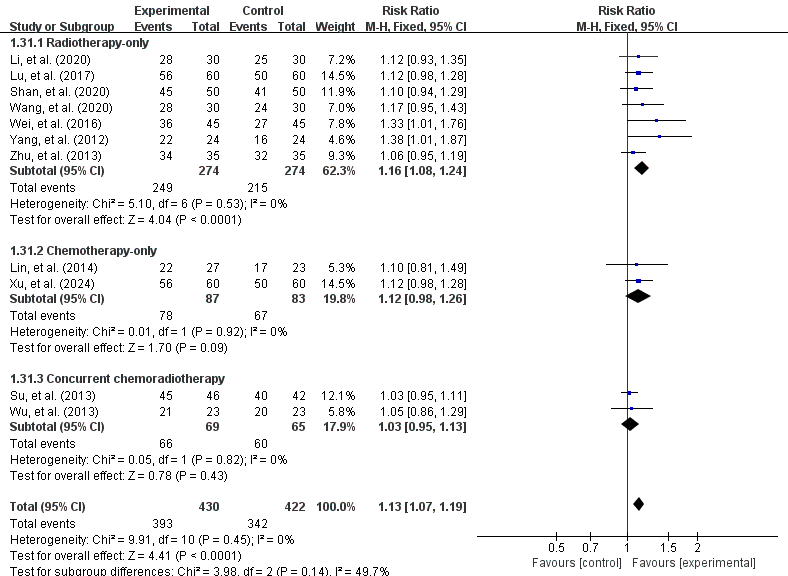


**Figure S1 (d):** Subgroup analysis of DCR (Control group treatment regimen).


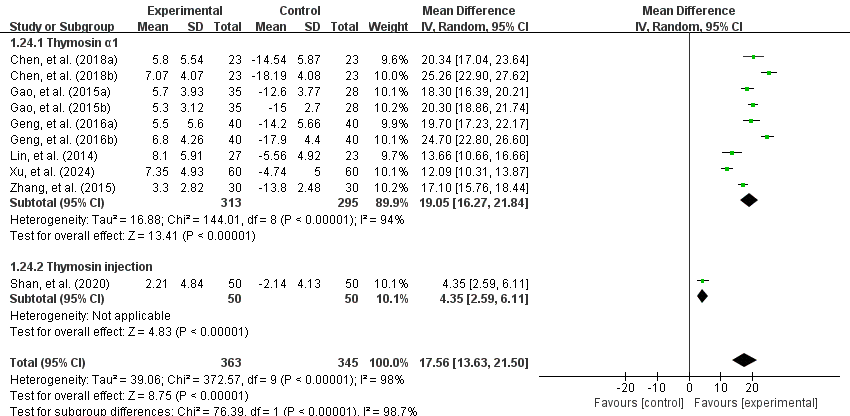


**Figure S1 (e):** Subgroup analysis of CD3^+^% (Thymosin type).


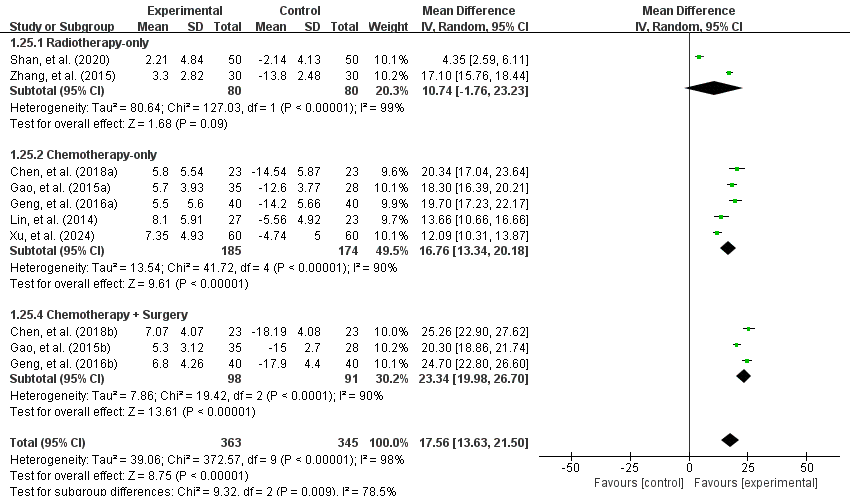


**Figure S1 (f):** Subgroup analysis of CD3^+^% (Control group treatment regimen).


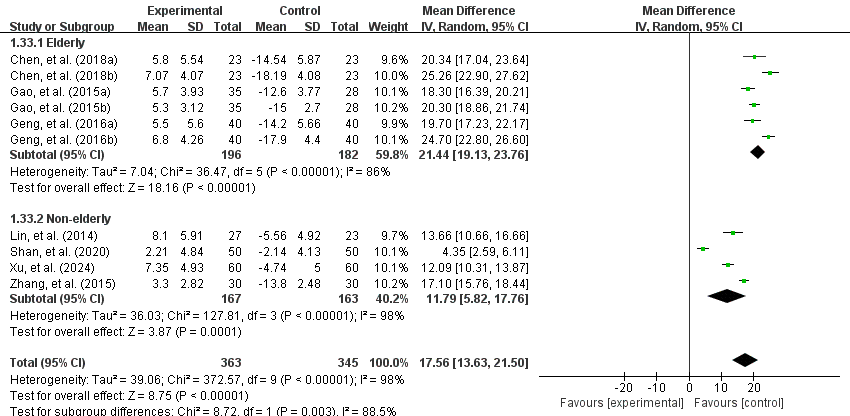


**Figure S1 (g):** Subgroup analysis of CD3^+^% (patient age).


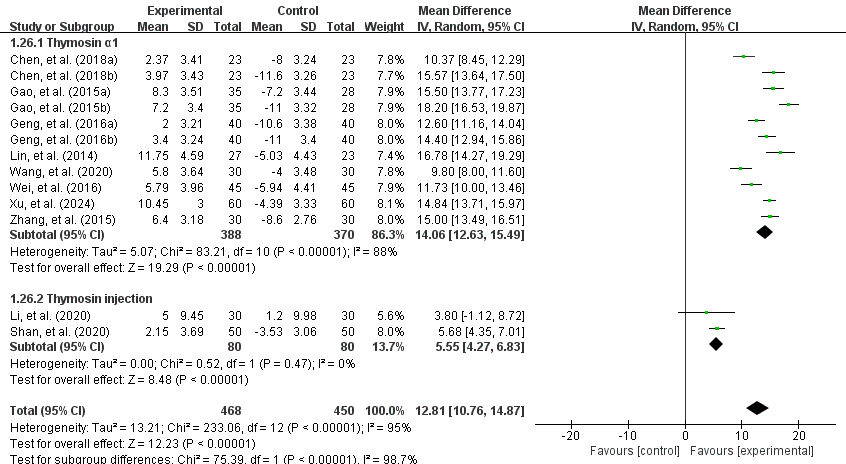


**Figure S1 (h):** Subgroup analysis of CD4^+^% (Thymosin type).


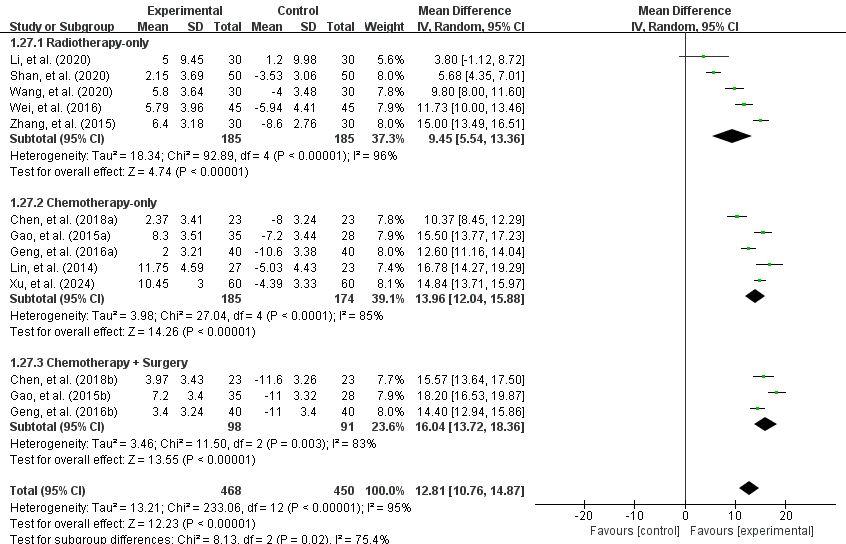


**Figure S1 (i):** Subgroup analysis of CD4^+^% (Control group treatment regimen).


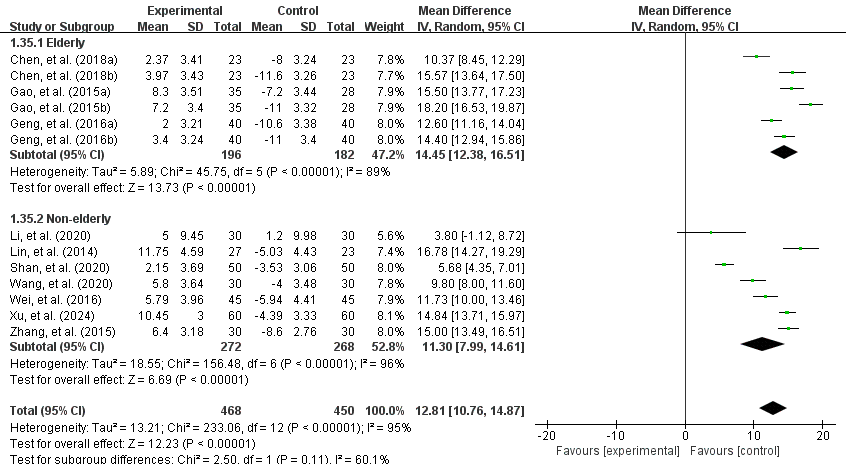


**Figure S1 (j):** Subgroup analysis of CD4^+^% (patient age).


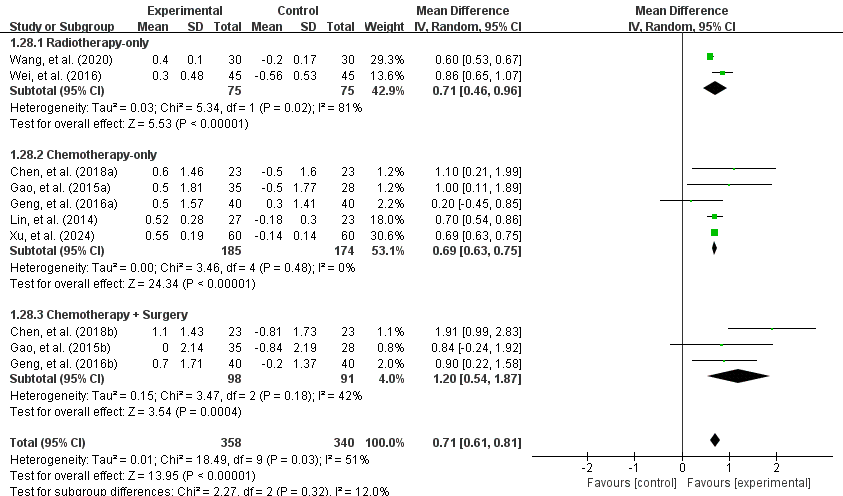


**Figure S1 (k):** Subgroup analysis of CD4^+^/CD8^+^ (Control group treatment regimen).


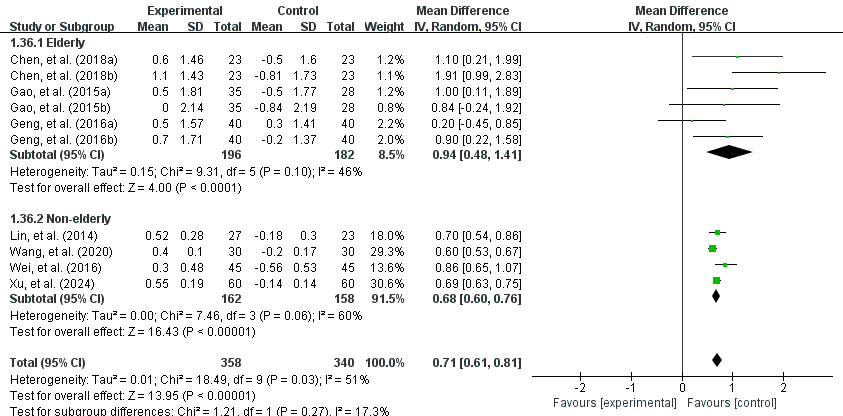


**Figure S1 (l):** Subgroup analysis of CD4^+^/CD8^+^ (patient age).


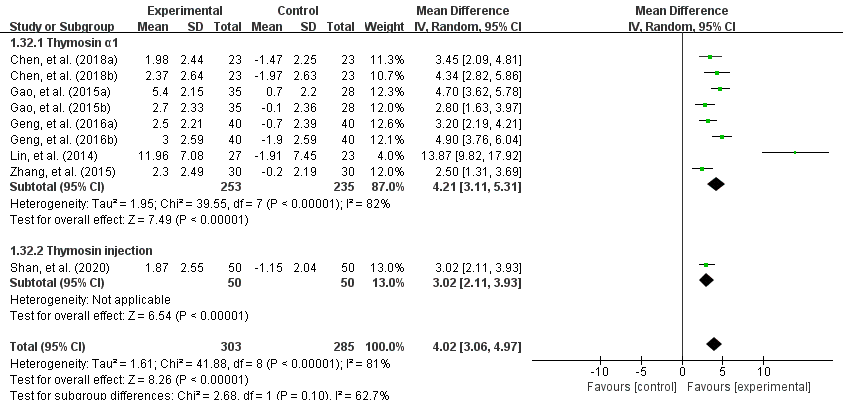


**Figure S1 (m):** Subgroup analysis of NK (Thymosin type).


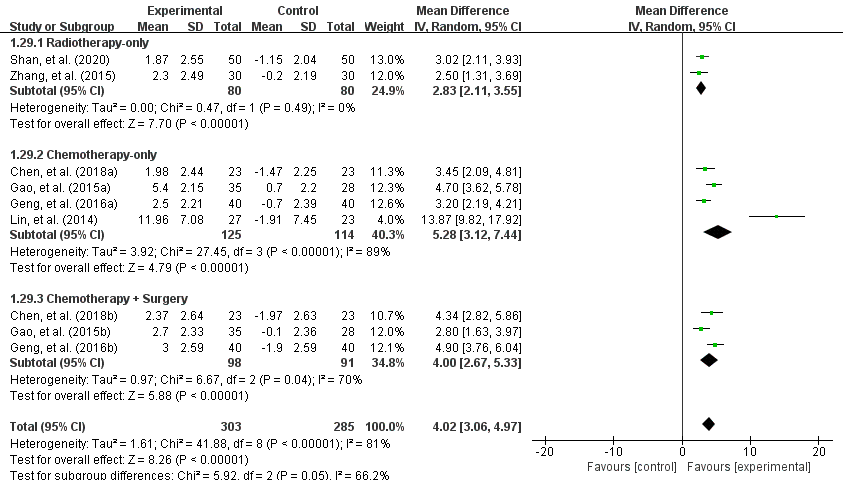


**Figure S1 (n):** Subgroup analysis of NK (Control group treatment regimen).


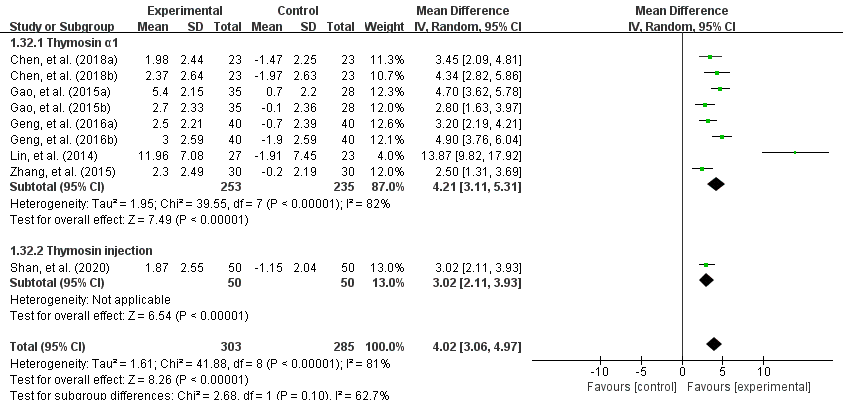


**Figure S1 (o):** Subgroup analysis of NK (patient age).

**Figure S1:** Subgroup analysis.

**Table S1:** Meta-regression analysis

| Outcomes | Meta-regression analysis | | |
| --- | --- | --- | --- |
|  | Thymosin type | Control group treatment regimen | Patient age (elderly vs. non-elderly) |
| CD3^+^% | P = 0.029 | P = 0.034 | P = 0.006 |
| CD4^+^% | P = 0.005 | P = 0.060 | P = 0.185 |
| CD4^+^/ CD8^+^ | - | P = 0.271 | P = 0.368 |
| NK | P = 0.660 | P = 0.887 | P = 0.504 |


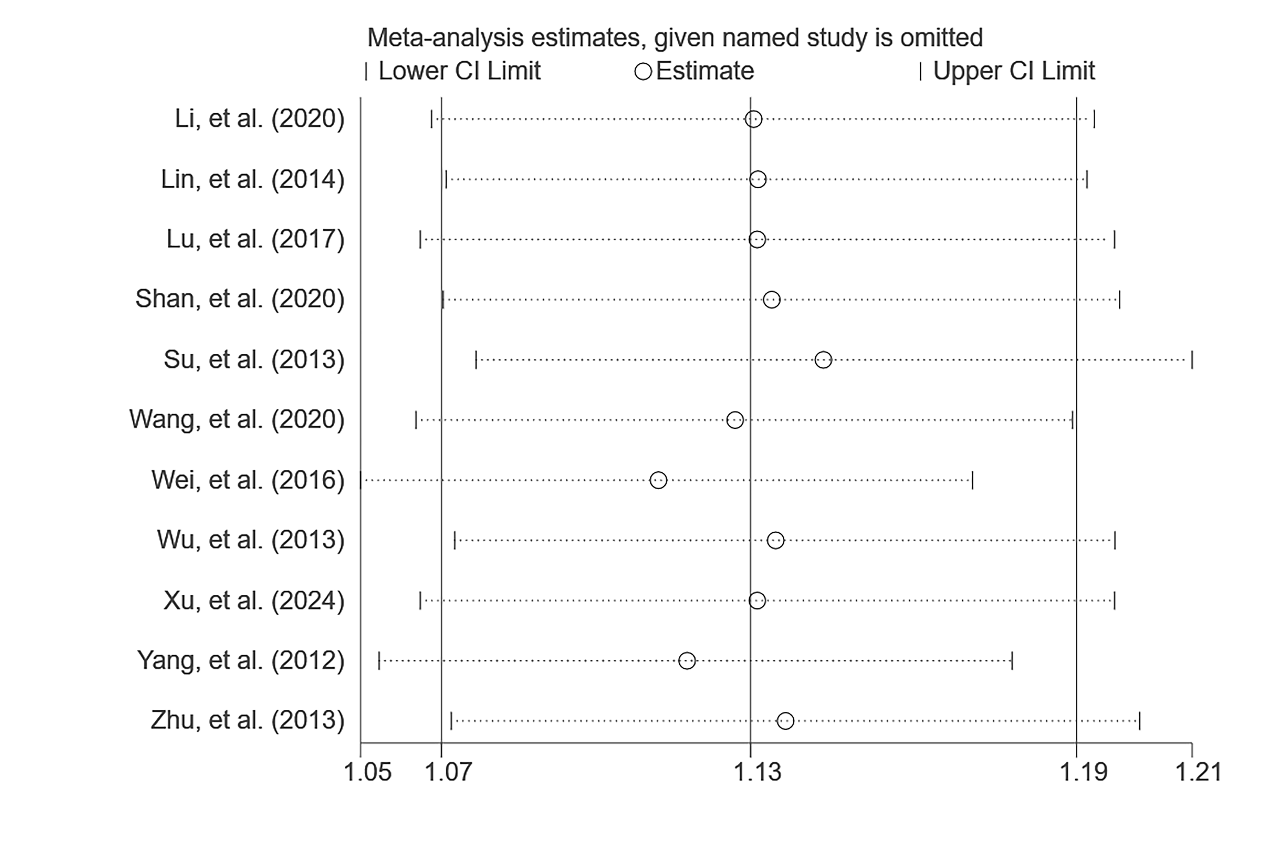


**Figure S2 (a)**


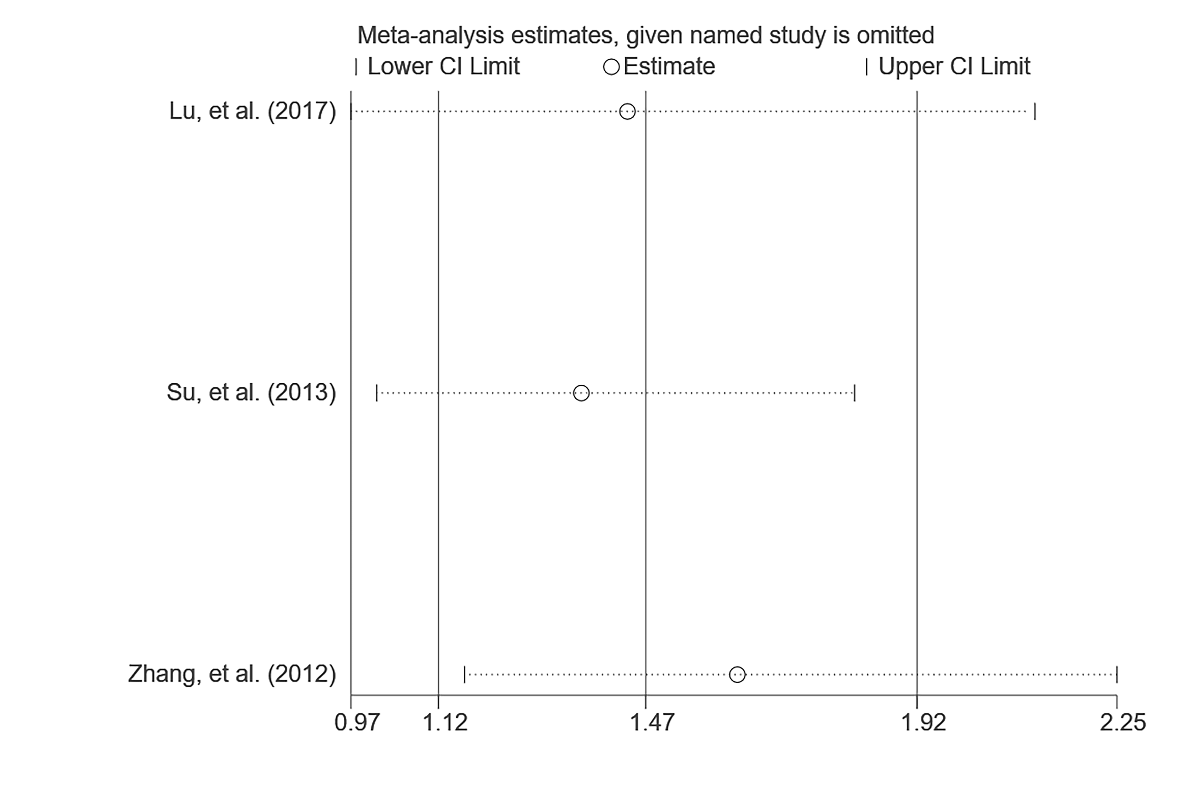


**Figure S2 (b)**


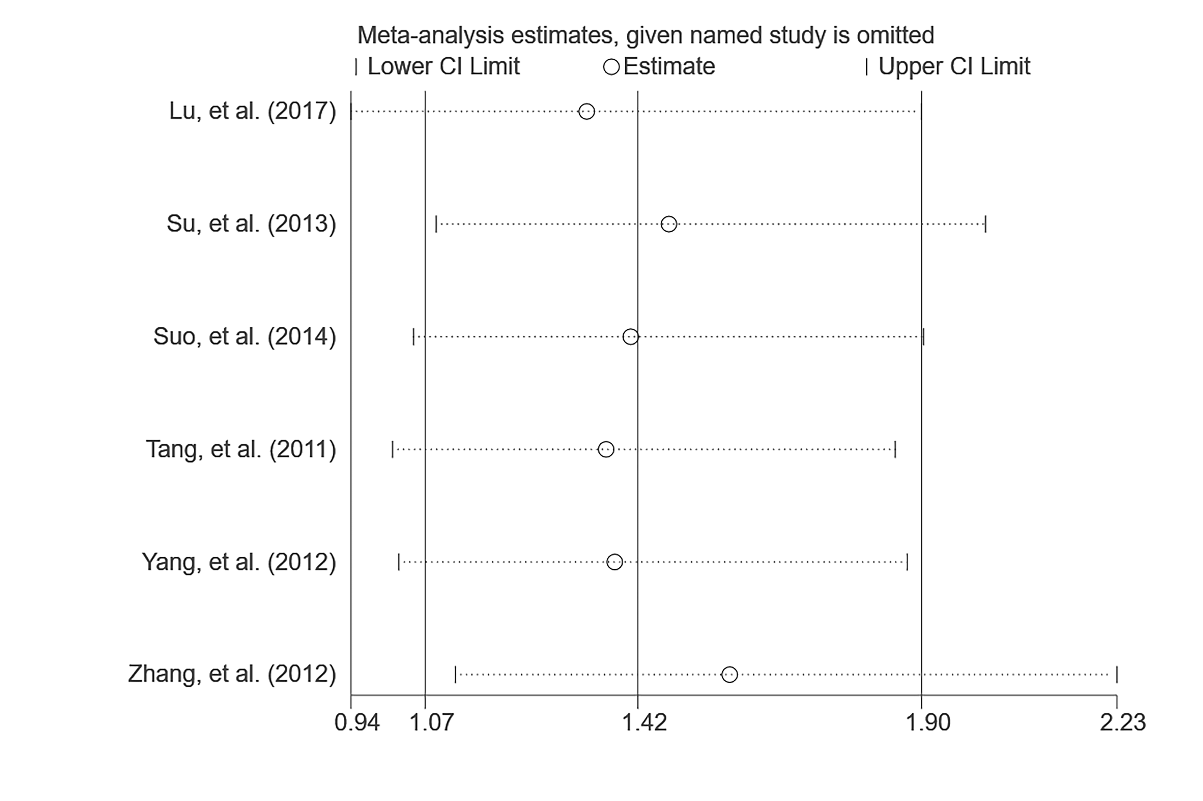


**Figure S2 (c)**


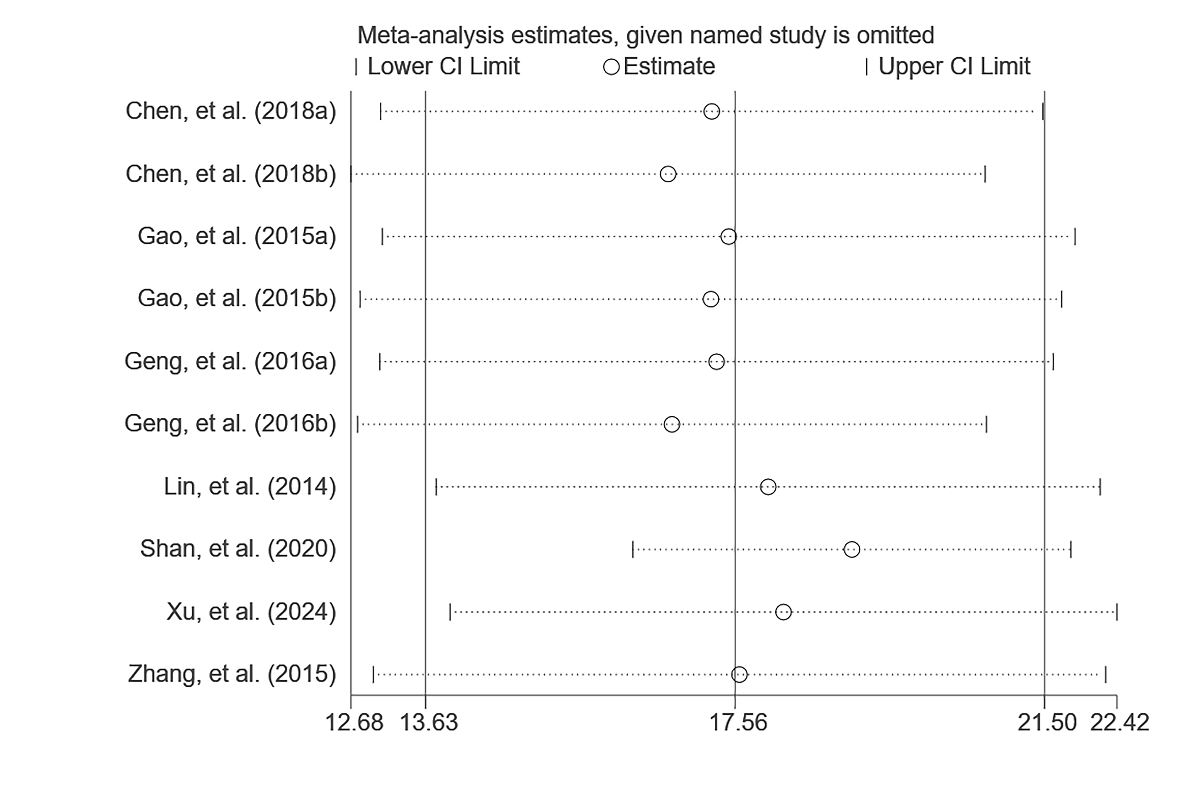


**Figure S2 (d)**


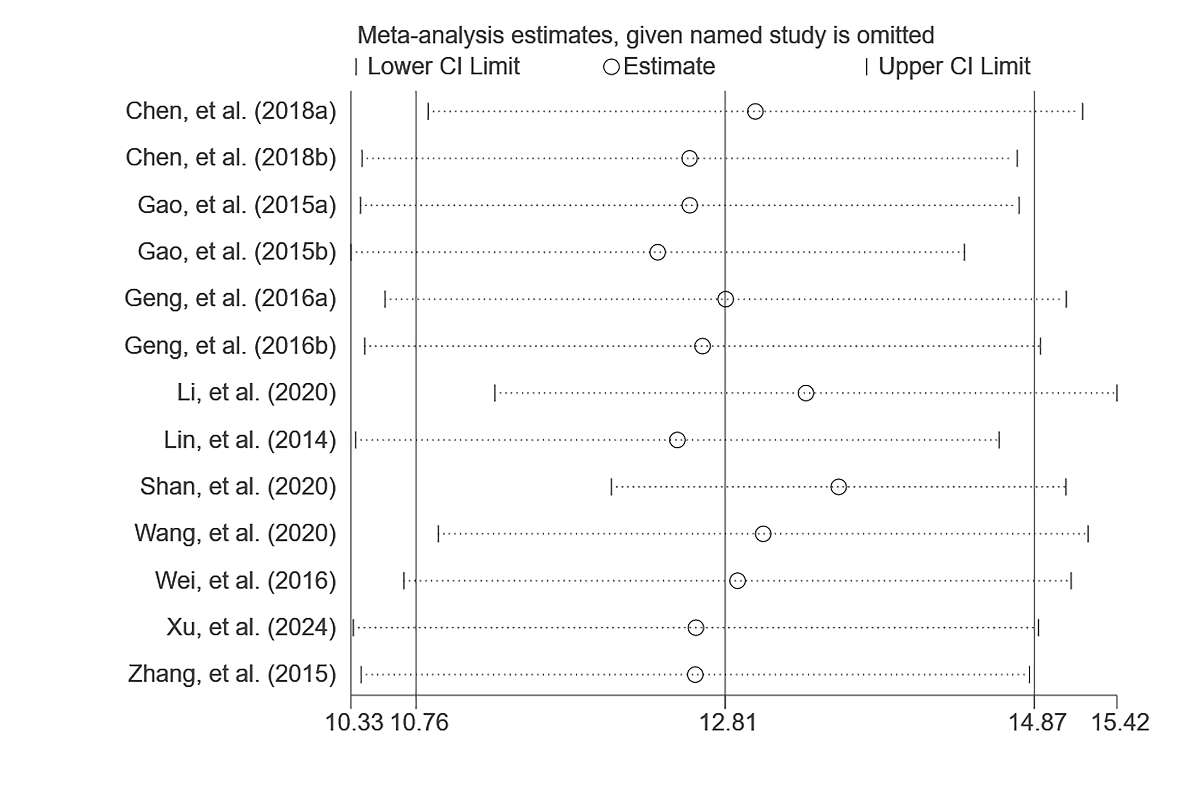


**Figure S2 (e)**


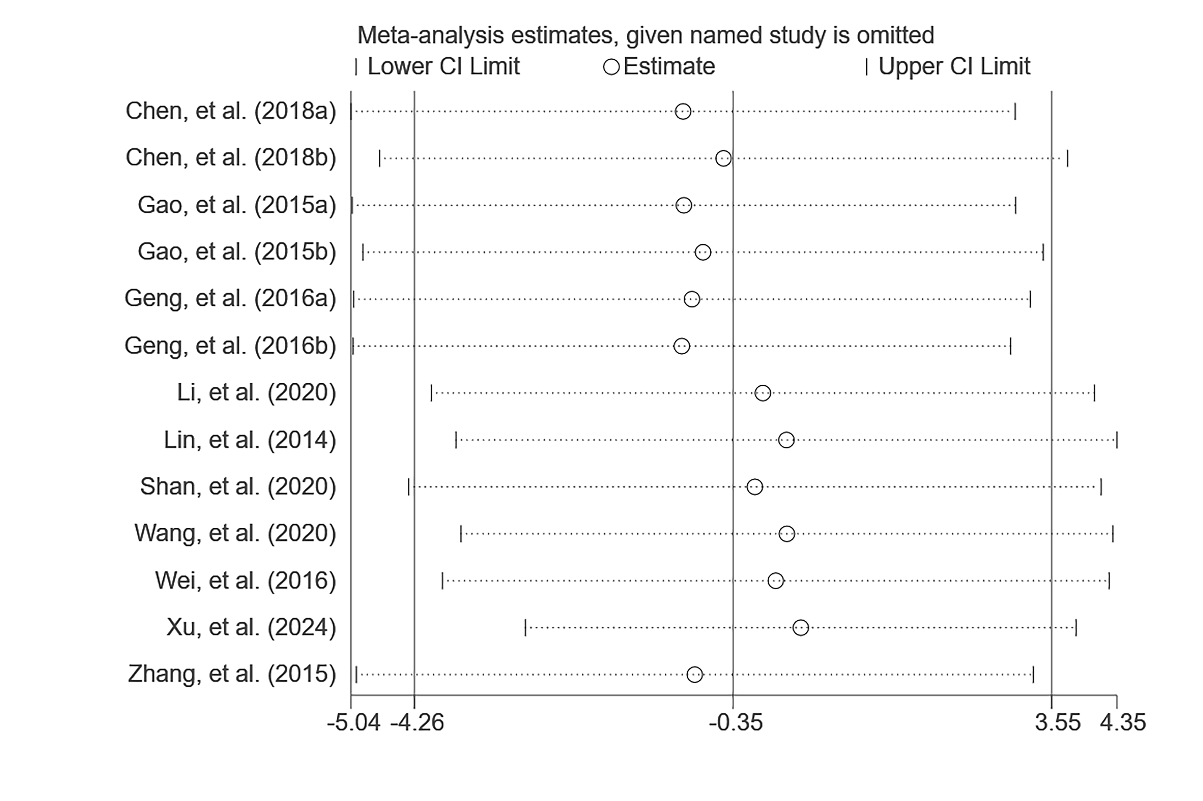


**Figure S2 (f)**


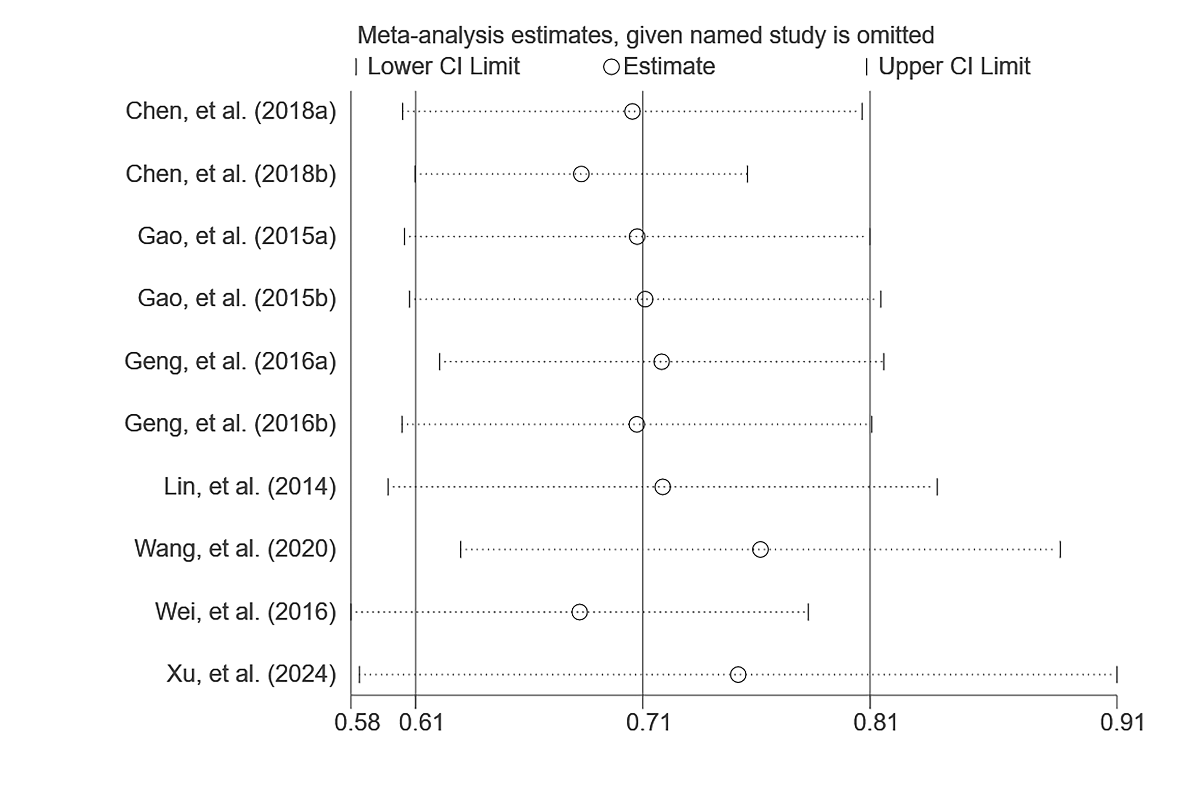


**Figure S2 (g)**


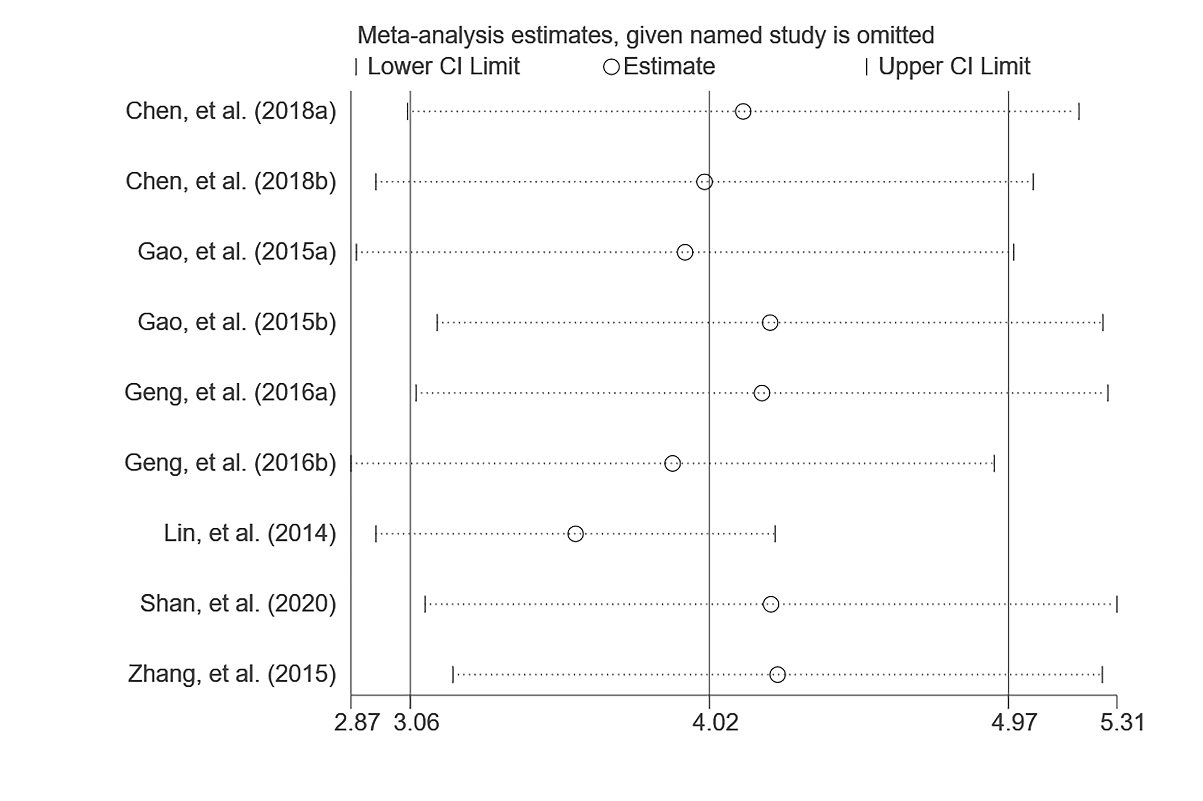


**Figure S2 (h)**


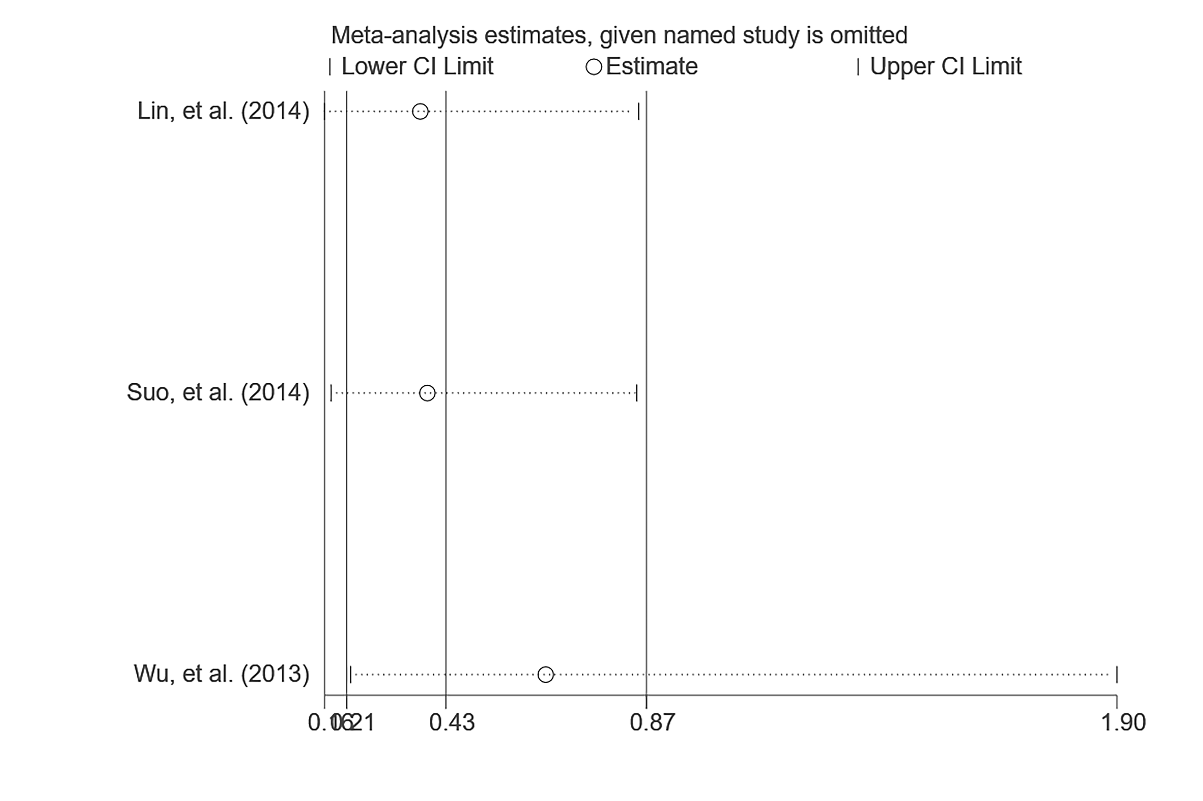


**Figure S2 (i)**


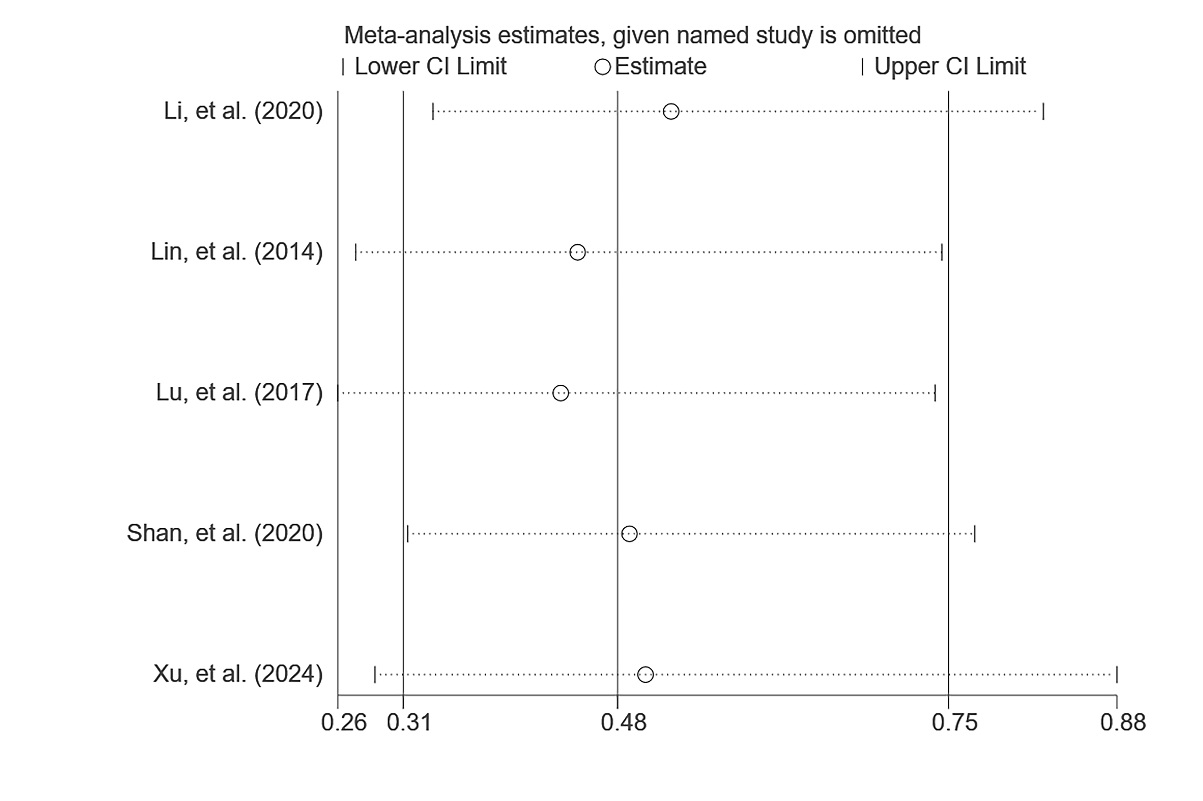


**Figure S2 (j)**


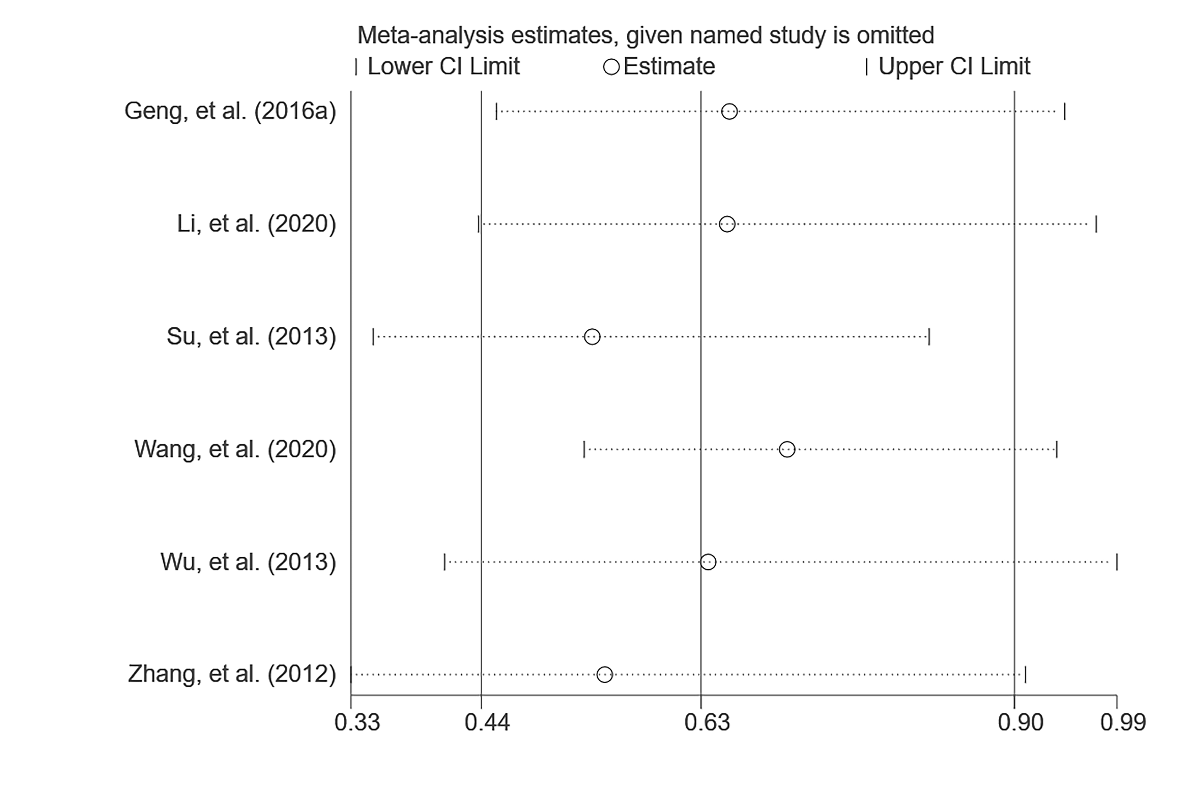


**Figure S2 (l)**


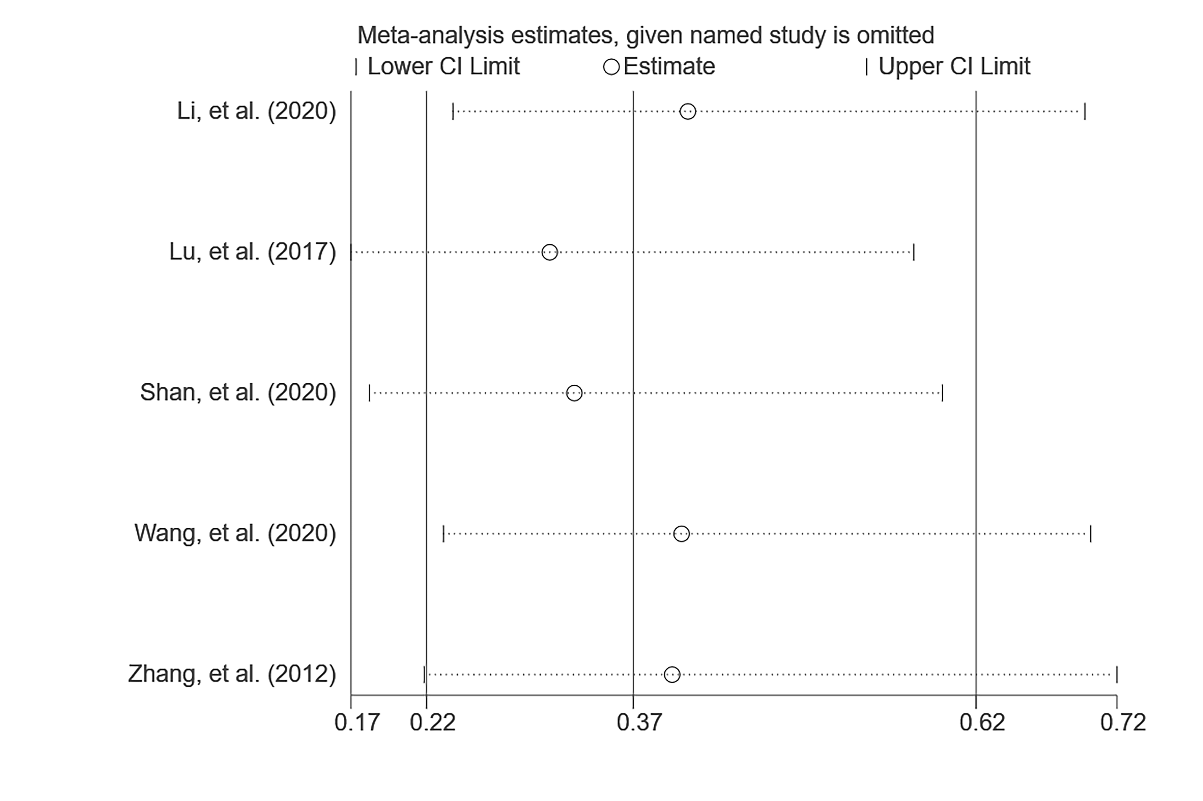


**Figure S2 (m)**

**Figure S2:** Sensitivity analyses: (a) DCR; (b) 3-year survival rate; (c) 3-year survival rate; (d) CD3^+^%; (e) CD4^+^%; (f) CD8^+^%; (g) CD4^+^/CD8^+^; (h) NK; (i) Thrombocytopenia; (j) Myelosuppression; (l) Radiation esophagitis; (m) Radiation pneumonitis.


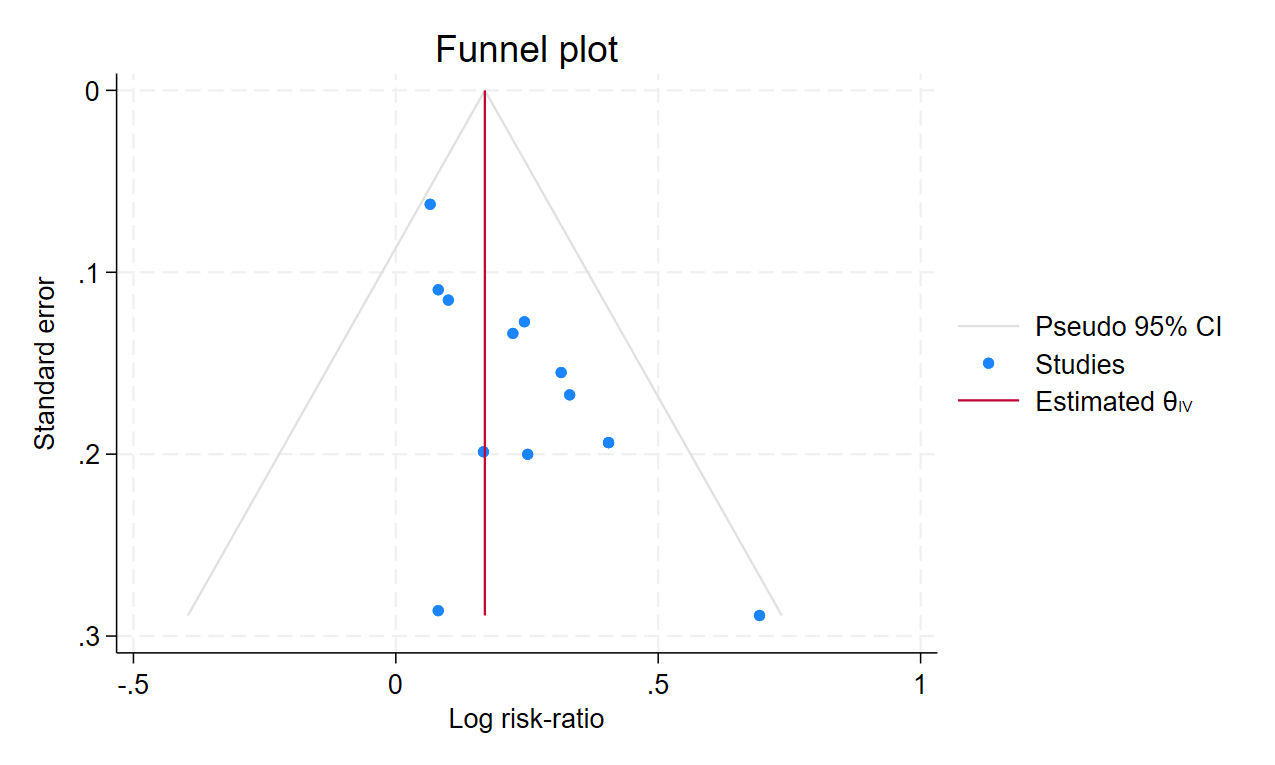


**Figure S3 (a)**


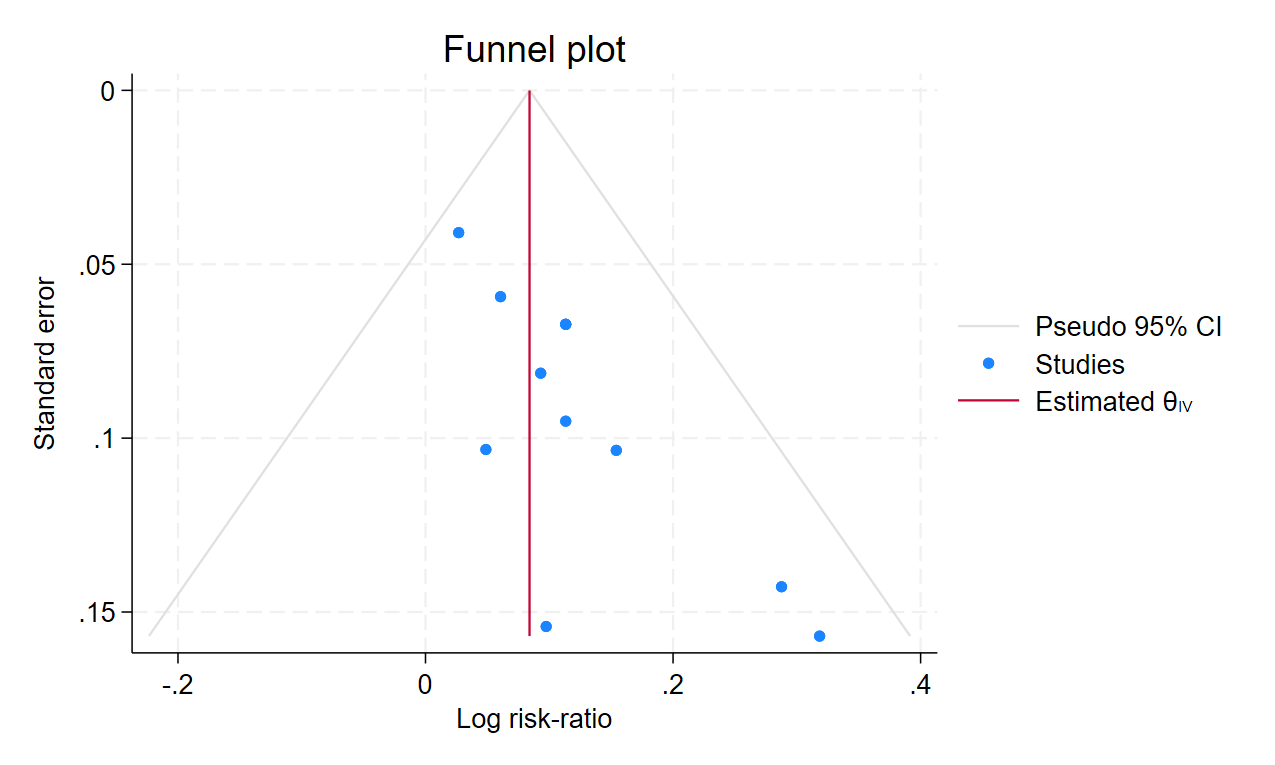


**Figure S3 (b)**


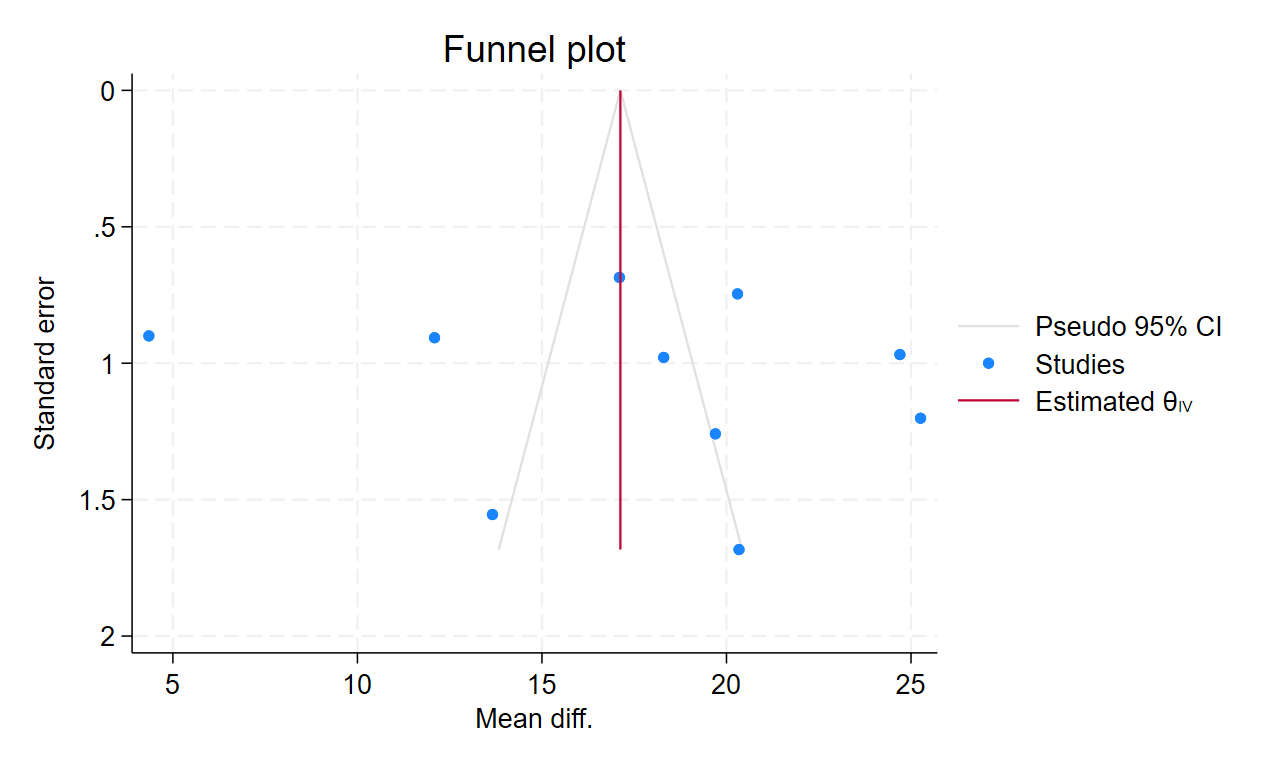


**Figure S3 (c)**


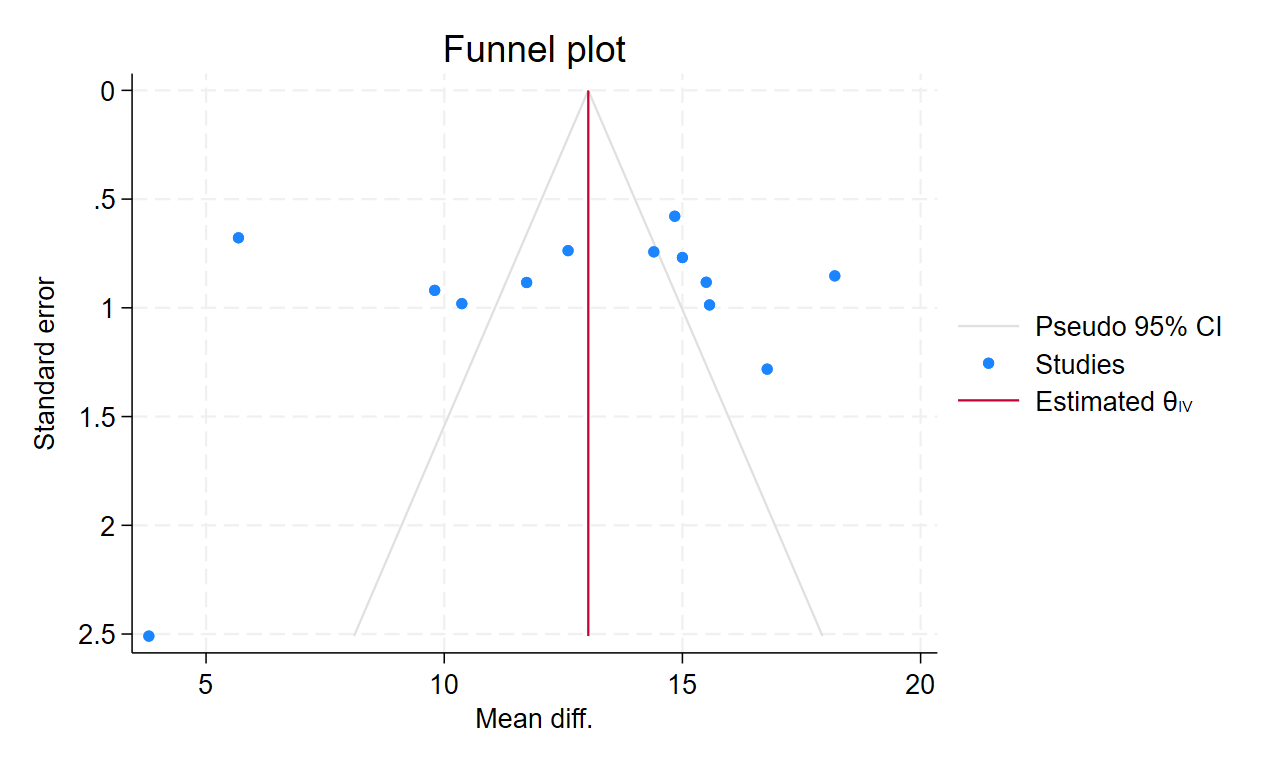


**Figure S3 (d)**


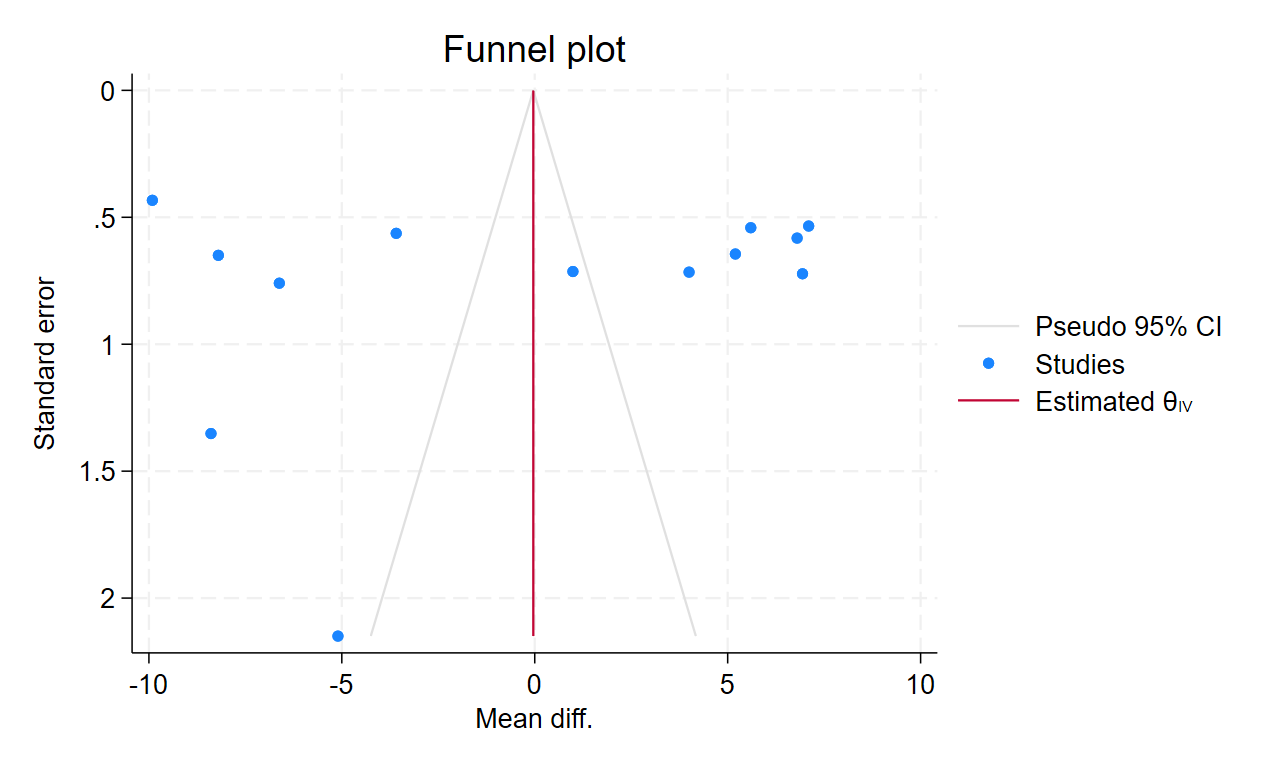


**Figure S3 (e)**


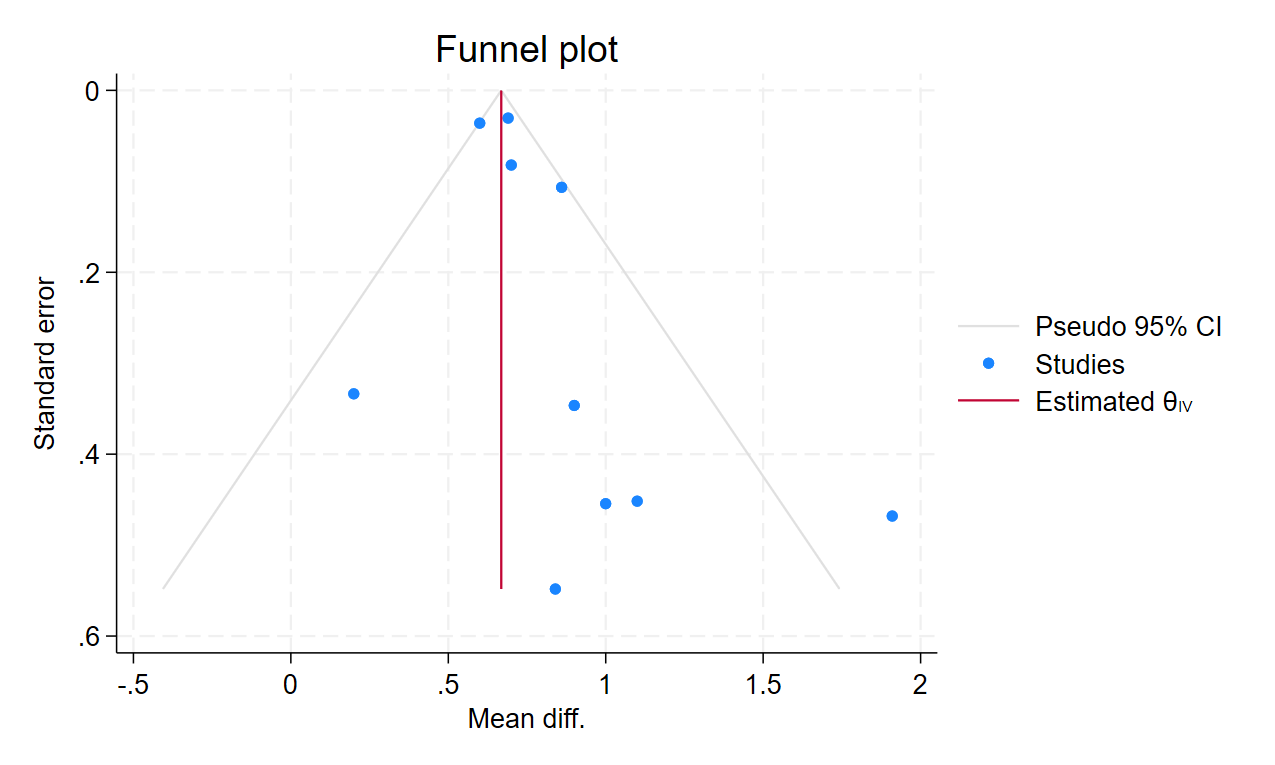


**Figure S3 (f)**


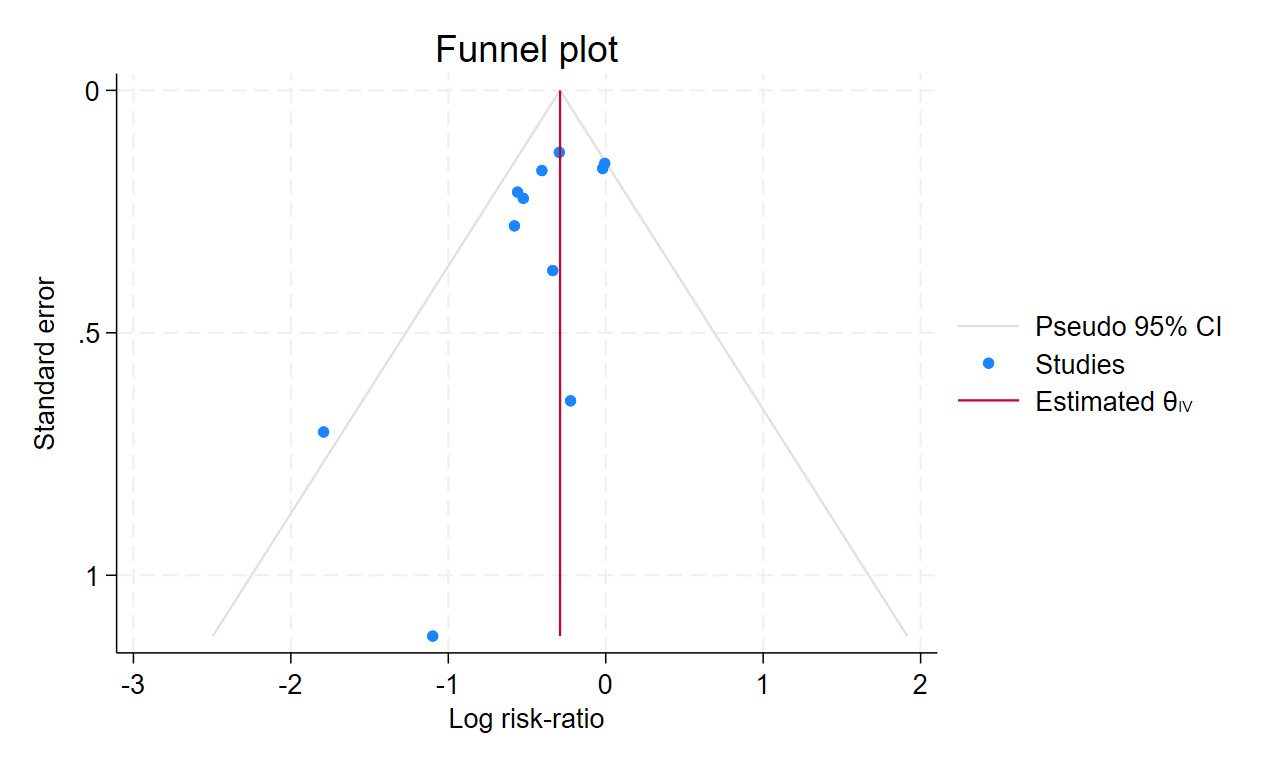


**Figure S3 (g)**


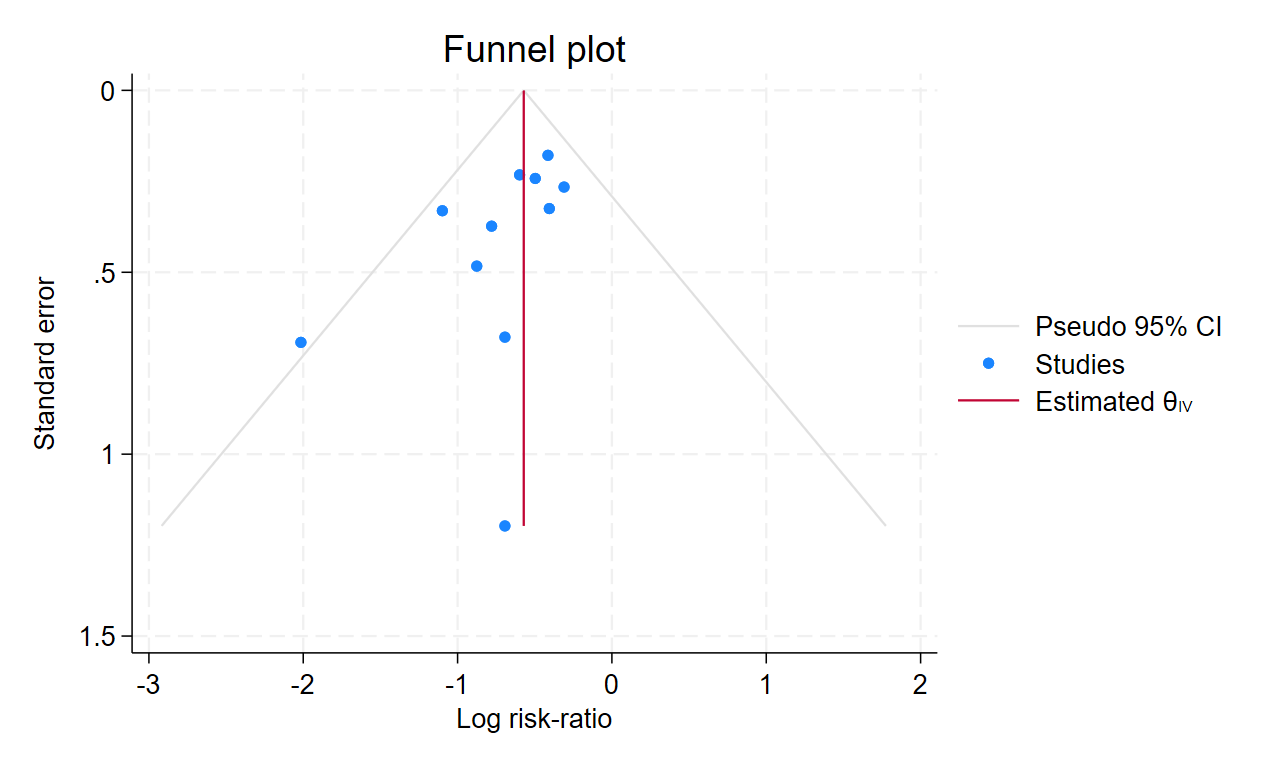


**Figure S3 (h)**

**Figure S3:** Funnel plots: (a) ORR; (b) DCR; (c) CD3^+^%; (d) CD4^+^%; (e) CD8^+^%; (f) CD4^+^/ CD8^+^; (g) gastrointestinal reactions; (h) leukopenia.
