# Supplementary material for: Efficacy and safety of thymosin combined with anticancer therapy for esophageal cancer: a systematic review and meta-analysis of randomized controlled trials
Source: Front Immunol. 2026 May 29;17:1812375. doi: 10.3389/fimmu.2026.1812375 (PMC13260294; doi:10.3389/fimmu.2026.1812375)
Supplement: Supplementary file 2 [file Table2.docx]

| Outcome and follow-up | Patients (studies), N | Relative effect (95% CI) | **Absolute effects (95% CI)** | | | Certainty |
| --- | --- | --- | --- | --- | --- | --- |
|  |  |  | **Control** | **Thymosin** | **Difference** |  |
| ORR | 965 (13 RCTs) | **RR = 1.27** (1.17 to 1.39) | 594 per 1,000 | **755 per 1,000** (826 to 695) | **160 more per 1,000** (from 101 more to 232 more) | ⨁⨁◯◯ Low^a,b^ |
| DCR | 852 (11 RCTs) | **RR = 1.13** (1.07 to 1.19) | 810 per 1,000 | **916 per 1,000** (964 to 867) | **105 more per 1,000** (from 57 more to 154 more) | ⨁⨁◯◯ Low^a,b^ |
| 1-year survival rate | 611 (8 RCTs) | **RR = 1.36** (1.19 to 1.56) | 488 per 1,000 | **664 per 1,000** (762 to 581) | **176 more per 1,000** (from 93 more to 274 more) | ⨁⨁◯◯ Low^a,c^ |
| 2-year survival rate | 275 (3 RCTs) | **RR = 1.47** (1.12 to 1.92) | 363 per 1,000 | **534 per 1,000** (697 to 407) | **171 more per 1,000** (from 44 more to 334 more) | ⨁⨁◯◯ Low^a,c^ |
| 3-year survival rate | 421 (6 RCTs) | **RR = 1.42** (1.07 to 1.90) | 255 per 1,000 | **362 per 1,000** (484 to 273) | **107 more per 1,000** (from 18 more to 229 more) | ⨁⨁◯◯ Low^a,c^ |
| KPS improvement rate | 208 (2 RCTs) | **RR = 1.39** (1.17 to 1.64) | 627 per 1,000 | **872 per 1,000** (1,000 to 734) | **245 more per 1,000** (from 107 more to 402 more) | ⨁⨁◯◯ Low^a,c^ |
| **CI:** confidence interval; **RR:** risk ratio | | | | | | |

a. Downgraded by one level: None of the studies mentioned the implementation of allocation concealment and blinding methods.

b. Downgrade by one level: Egger's test p < 0.05.

c. Downgraded by one level: Insufficient number of studies reporting outcomes, n < 10.

| Outcome and follow-up | Patients (studies), N | Relative effect (95% CI) | **Absolute effects (95% CI)** | | | Certainty |
| --- | --- | --- | --- | --- | --- | --- |
|  |  |  | **Control** | **Thymosin** | **Difference** |  |
| CD3⁺% | (9 RCTs) | - | 0 | - | **17.56** (13.63 to 21.5) | ⨁◯◯◯ Very low^a,b,c^ |
| CD4⁺% | (13 RCTs) | - | 0 | - | **12.81** (10.76 to 14.87) | ⨁◯◯◯ Very low^a,b^ |
| CD8⁺% | (13 RCTs) | - | 0 | - | **-0.35** (-4.26 to 3.55) | ⨁◯◯◯ Very low^a,b,d^ |
| CD4⁺/CD8⁺ | (10 RCTs) | - | 0 | - | **0.71** (0.61 to 0.81) | ⨁⨁◯◯ Low^a,e^ |
| NK | (9 RCTs) | - | 0 | - | **4.02** (3.06 to 4.97) | ⨁◯◯◯ Very low^a,b,c^ |
| **CI:** confidence interval; **MD:** mean difference | | | | | | |

a. Downgraded by one level: None of the studies mentioned the implementation of allocation concealment and blinding methods.

b. Downgraded by two level: I² > 75%.

c. Downgraded by one level: Insufficient number of studies reporting outcomes, n < 10.

d. Downgrade by one level: The 95% confidence interval for the pooled effect size spans the boundary of non-significance.

e. Downgraded by one level: I² > 50%.

| Outcome and follow-up | Patients (studies), N | Relative effect (95% CI) | **Absolute effects (95% CI)** | | | Certainty |
| --- | --- | --- | --- | --- | --- | --- |
|  |  |  | **Control** | **Thymosin** | **Difference** |  |
| Gastrointestinal reactions | 819 (11 RCTs) | **RR = 0.69** (0.60 to 0.79) | 495 per 1,000 | **342 per 1,000** (391 to 297) | **153 fewer per 1,000** (from 198 fewer to 104 fewer) | ⨁⨁◯◯ Low^a,b^ |
| Leukopenia | 769 (11 RCTs) | **RR = 0.52** (0.43 to 0.63) | 456 per 1,000 | **237 per 1,000** (287 to 196) | **219 fewer per 1,000** (from 260 fewer to 169 fewer) | ⨁⨁⨁◯ Moderate^a^ |
| Thrombocytopenia | 142 (3 RCTs) | **RR = 0.43** (0.21 to 0.87) | 290 per 1,000 | **125 per 1,000** (252 to 61) | **165 fewer per 1,000** (from 229 fewer to 38 fewer) | ⨁⨁◯◯ Low^a,c^ |
| Myelosuppression | 450 (5 RCTs) | **RR = 0.48** (0.31 to 0.75) | 224 per 1,000 | **108 per 1,000** (168 to 70) | **117 fewer per 1,000** (from 155 fewer to 56 fewer) | ⨁⨁◯◯ Low^a,c^ |
| Radiation oesophagitis | 401 (6 RCTs) | **RR = 0.63** (0.44 to 0.90) | 540 per 1,000 | **340 per 1,000** (486 to 238) | **200 fewer per 1,000** (from 303 fewer to 54 fewer) | ⨁◯◯◯ Very low^a,c,d^ |
| Radiation pneumonitis | 407 (5 RCTs) | **RR = 0.37** (0.22 to 0.62) | 236 per 1,000 | **87 per 1,000** (147 to 52) | **149 fewer per 1,000** (from 184 fewer to 90 fewer) | ⨁⨁◯◯ Low^a,c^ |
| **CI:** confidence interval; **RR:** risk ratio | | | | | | |

a. Downgraded by one level: None of the studies mentioned the implementation of allocation concealment and blinding methods.

b. Downgrade by one level: Egger's test p < 0.05.

c. Downgraded by one level: Insufficient number of studies reporting outcomes, n < 10.

d. Downgraded by one level: I² > 50%.

| Outcome and follow-up | Patients (studies), N | Relative effect (95% CI) | **Absolute effects (95% CI)** | | | Certainty |
| --- | --- | --- | --- | --- | --- | --- |
|  |  |  | **Control** | **Thymosin** | **Difference** |  |
| Postoperative pulmonary infection | 126 (2 RCTs) | **RR = 0.20** (0.06 to 0.66) | 238 per 1,000 | **48 per 1,000** (157 to 14) | **190 fewer per 1,000** (from 224 fewer to 81 fewer) | ⨁⨁◯◯ Low^a,b^ |
| Postoperative anastomotic fistula | 126 (2 RCTs) | **RR = 0.40** (0.08 to 1.98) | 79 per 1,000 | **32 per 1,000** (157 to 6) | **48 fewer per 1,000** (from 73 fewer to 78 more) | ⨁◯◯◯ Very low^a,b,c^ |
| Postoperative arrhythmia | 126 (2 RCTs) | **RR = 2.00** (0.38 to 10.50) | 32 per 1,000 | **63 per 1,000** (333 to 12) | **32 more per 1,000** (from 20 fewer to 302 more) | ⨁◯◯◯ Very low^a,b,c^ |
| Postoperative atelectasis | 126 (2 RCTs) | **RR = 1.00** (0.26 to 3.84) | 63 per 1,000 | **63 per 1,000** (244 to 17) | **0 fewer per 1,000** (from 47 fewer to 180 more) | ⨁◯◯◯ Very low^a,b,c^ |
| **CI:** confidence interval; **RR:** risk ratio | | | | | | |

a. Downgraded by one level: None of the studies mentioned the implementation of allocation concealment and blinding methods.

b. Downgraded by one level: Insufficient number of studies reporting outcomes, n < 10.

c. Downgrade by one level: The 95% confidence interval for the pooled effect size spans the boundary of non-significance.
